# Spatial Transcriptional Dynamics of CD74⁺ B Cells in Tertiary Lymphoid Structures Drive Immune Evolution in Penile Squamous Cell Carcinoma

**DOI:** 10.1002/advs.202509742

**Published:** 2025-10-20

**Authors:** Ting Xue, Chuangzhong Deng, Jingya Liu, Ru Yan, Jing Li, Xiheng Hu, Xueying Li, Xiao Xiao, Jietian Jin, Hongzhen Tang, Desi Chen, Zihan Zuo, Yujie Liang, Dongbin Wang, Bonan Chen, Hui Han, Zaishang Li

**Affiliations:** ^1^ State Key Laboratory of Oncology in South China Guangdong Provincial Clinical Research Center for Cancer Sun Yat‐sen University Cancer Center Guangzhou 510060 China; ^2^ Department of Urology Sun Yat sen University Cancer Center Guangzhou 510060 China; ^3^ Department of Musculoskeletal Oncology Sun Yat‐sen University Cancer Center Guangzhou 510060 China; ^4^ BioMega Spatiotemporal Pathology Research Center Zhuhai 519000 China; ^5^ Department of Oncology Guizhou Provincial People's Hospital Guizhou 550008 China; ^6^ Department of Urology Cancer Hospital Affiliated to Guangzhou Medical University Guangzhou 510030 China; ^7^ Department of Urology Xiangya Hospital Central South University Changsha 410008 China; ^8^ Department of Dermatology Xiangya Hospital Central South University Changsha 410008 China; ^9^ National Engineering Research Center of Personalized Diagnostic and Therapeutic Technology Changsha 410008 China; ^10^ Furong Laboratory Changsha 410008 China; ^11^ Department of Oncology The Seventh Affiliated Hospital Sun Yat‐sen University Shenzhen 518107 China; ^12^ Department of Pathology Sun Yat‐sen University Cancer Center Guangzhou 510060 China; ^13^ Department of Urology Shenzhen People's Hospital The Second Clinic Medical College of Jinan University Shenzhen 518020 China; ^14^ Clinical Medicine Guangzhou University of Chinese Medicine Guangzhou 510060 China; ^15^ Shenzhen Kangning Hospital Shenzhen 518020 China; ^16^ Dongguan Key Laboratory of Public Health Laboratory Science School of Public Health Guangdong Medical University Dongguan 523808 China; ^17^ Department of Anatomical and Cellular Pathology State Key Laboratory of Translational Oncology Sir Y. K. Pao Cancer Center Prince of Wales Hospital The Chinese University of Hong Kong Hong Kong China; ^18^ Institute of Digestive Disease State Key Laboratory of Digestive Disease Li Ka Shing Institute of Health Science The Chinese University of Hong Kong Hong Kong China; ^19^ CUHK‐Shenzhen Research Institute Shenzhen 518020 China; ^20^ Collaborative Innovation Center of Cancer Medicine Guangzhou 510060 China; ^21^ Department of Urology First Affiliated Hospital of Southern University of Science and Technology Shenzhen 518020 China; ^22^ Shenzhen Clinical Research Center for Geriatrics Shenzhen People's Hospital Shenzhen 518020 China

**Keywords:** CD74⁺ B cells, immunotherapy target, penile squamous cell carcinoma, spatial transcriptomics, tertiary lymphoid structures

## Abstract

Penile squamous cell carcinoma (PSCC) is a malignancy characterized by a poor prognosis and lack of reliable biomarkers, presenting considerable therapeutic challenges. Tertiary lymphoid structures (TLSs) are critical modulators of antitumor immunity; however, the immunological dynamics, particularly the roles of B cells and their interactions with naive T cells in PSCC tissues, remain inadequately understood. This study integrates transcriptomic approaches, including spatial transcriptomics, single‐cell sequencing (scRNA‐seq), and bulk RNA sequencing (bulk RNA‐seq), and immunohistochemistry to elucidate the immune architecture of and functional mechanisms within TLSs. The results reveal a positive correlation between TLS density and patient survival, with CD74⁺ B cells tending to be enriched during early TLS formation. These cells exhibit strong immune activation and a propensity to differentiate into plasma cells. By engaging with naive T cells through *HLA‐DRA* via ligand–receptor interactions, CD74⁺ B cells activate transcription factors, including *NFKB1*, *NFKB2*, *NFATC1*, *NFATC2*, *FOS*, and *RUNX1*, in naive T cells, thereby enhancing the immune response. Consequently, CD74⁺ B cells represent a compelling biomarker for and therapeutic target of PSCC, offering profound insights into the immunological mechanisms that drive PSCC progression and response to immunotherapy.

## Graphical abstract

In penile squamous cell carcinoma, a higher tertiary lymphoid structure score is associated with improved survival. This is mediated by CD74⁺ B cells, which exhibit early developmental plasticity and the ability to differentiate into plasma cells. Functioning as a molecular switch, these cells activate naive T cells via *HLA‐DRA*–CD4 interaction, thereby initiating a robust antitumor immune response.

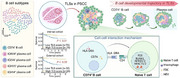

## 1 Introduction

Penile squamous cell carcinoma (PSCC) is an increasingly critical public health issue, driven by its increasing incidence and poor prognosis.^[^ ^1^, ^2^ ^]^ Despite available treatments, including surgery, radiation, and systemic therapies, advanced‐stage patients face limited therapeutic options, leading to reductions in progression‐free survival (PFS) and overall survival (OS).^[^ ^3^, ^4^, ^5^, ^6^ ^]^ Immunotherapy, as an emerging treatment modality, holds considerable promise for PSCC treatment, particularly immune checkpoint inhibition and modulation of the tumor microenvironment.^[^ ^7^, ^8^ ^]^ However, the precise mechanisms of immune evasion in PSCC remain poorly understood, and the intricate interactions among the tumor and immune system require further investigation. Such insights are essential for improving the therapeutic efficacy of immunotherapy and addressing the underlying challenges of immune resistance in PSCC.

Notably, tertiary lymphoid structures (TLSs) play pivotal roles in modulating the efficacy of cancer immunotherapy.^[^ ^9^, ^10^ ^]^ These organized lymphoid aggregates form in response to chronic inflammation and tumorigenesis and function as critical players in adaptive immune responses.^[^ ^11^ ^]^ Recent clinical studies have shown that TLSs may significantly improve the prognosis of PSCC patients by facilitating T‐cell activation and bolstering antitumor immunity. Our previous research demonstrated that the presence of TLSs alters the PSCC tumor microenvironment, transitioning it from a nonlymphoid to a lymphoid metastatic state.^[^ ^12^ ^]^ This transition underscores the potential of TLSs in guiding therapeutic strategies. Within TLS regions, B cells hold a central position in immune surveillance and regulation.^[^ ^13^, ^14^, ^15^ ^]^ Traditionally known for their role in antibody production, B cells within TLSs are becoming increasingly recognized as paramount antigen‐presenting cells (APCs). Through interactions with other immune cells, B cells enhance antitumor immunity, particularly by initiating T‐cell responses. CD74, a molecular chaperone for major histocompatibility complex (MHC) class II molecules, is integral to the process of B‐cell differentiation.^[^ ^16^, ^17^ ^]^ By promoting antigen presentation and activating the NF‐κB signaling pathway via its interaction with the BAFF receptor, CD74 regulates B‐cell survival, proliferation, and antibody production.^[^ ^18^ ^]^ Moreover, CD74 functions as a coordinator for APCs, precisely regulating immune responses through its interactions with other immune cell populations. In the tumor microenvironment, CD74 also acts as a regulator of tumor‐infiltrating regulatory T cells (Tregs) by modulating their accumulation and function.^[^ ^19^, ^20^ ^]^ By interfering with Treg‐mediated immune suppression, CD74 represents a promising therapeutic target, providing a novel approach to enhance antitumor immunity in PSCC.

In the tumor immune microenvironment, naive T cells represent a reservoir of potential cytotoxic responses against tumor cells.^[^ ^21^ ^]^ The activation of naive T cells within TLSs is primarily mediated by antigen presentation through B cells,^[^ ^22^ ^]^ which subsequently differentiate into effector T cells capable of mounting robust immune responses.^[^ ^23^ ^]^ However, the mechanisms governing cell–cell interactions within the immune microenvironment of PSCC tumors remain poorly defined.

In this study, we employ spatial transcriptomics, single‐cell RNA sequencing (scRNA‐seq), and bulk RNA sequencing (bulk RNA‐seq) to characterize the cellular interactions within the tumor‐associated TLSs of PSCC comprehensively. Our findings reveal a significant positive correlation between TLS abundance and survival outcomes in PSCC patients. Notably, we identified a functionally distinct subset of B cells, CD74⁺ B cells, within TLSs. This subset is spatially enriched in TLS regions and exhibits pronounced immunostimulatory activity, along with transcriptional features indicative of plasma cell differentiation. Further analyses reveal that CD74⁺ B cells express *HLA‐DRA* and engage CD4⁺ naive T cells through specific ligand–receptor interactions, thereby facilitating T‐cell priming and functional polarization. These results indicate that CD74⁺ B cells act as both APCs and immune amplifiers within TLSs, bridging humoral and cellular immunity and contributing to the immune regulatory landscape of PSCC.

## 2 Results

### 2.1 A High Abundance of TLSs is Positively Correlated with a Favorable Prognosis in Patients with PSCC

**Figure 1:**
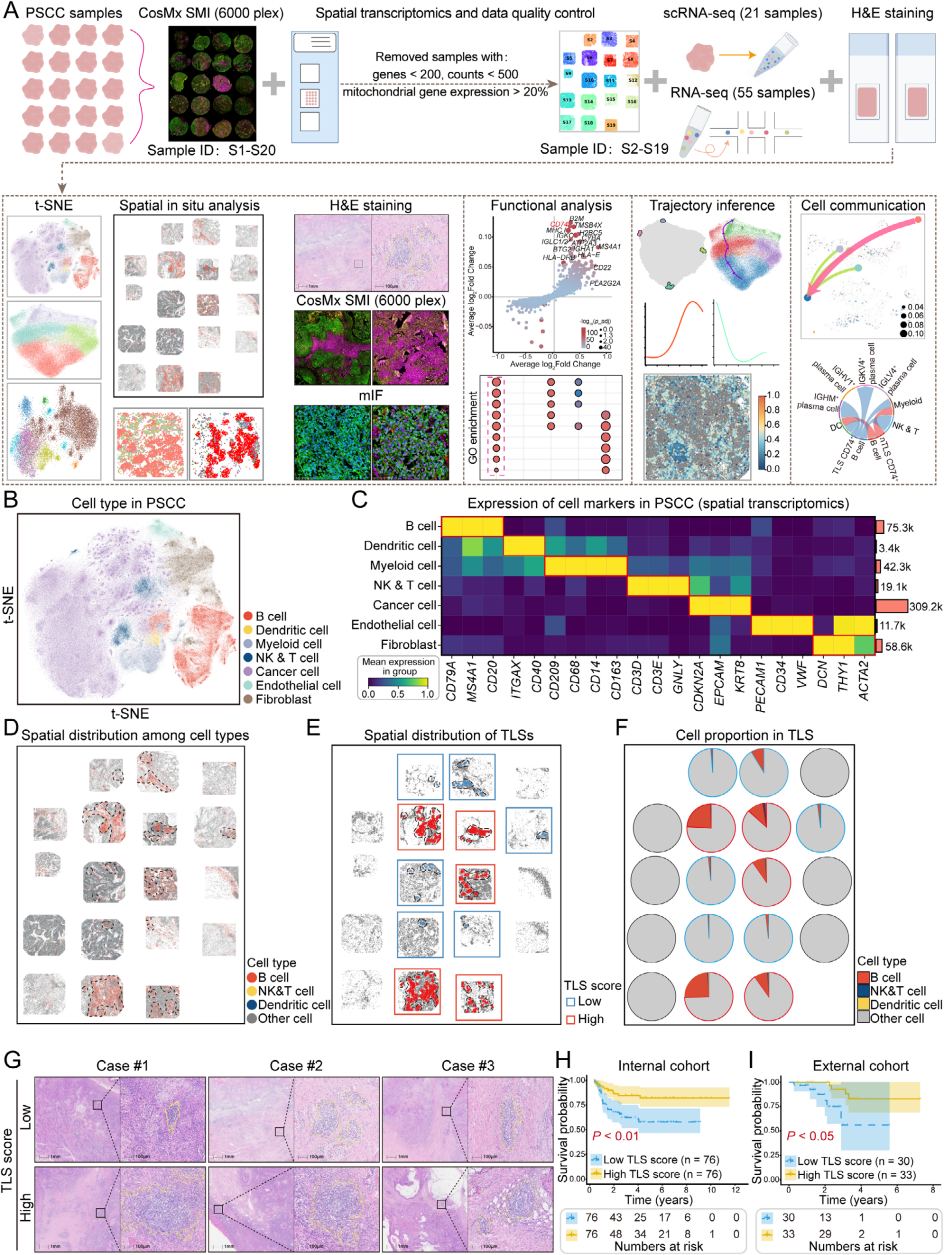
Immune landscape and prognostic significance of TLSs in the PSCC tumor microenvironment. A) Overview of the experimental workflow, including 18 PSCC samples analyzed by spatial transcriptomics, scRNA‐seq (*n* = 21), bulk RNA‐seq (*n* = 55), H&E staining, and mIF staining. Bioinformatics analyses, such as enrichment analysis, pseudotime trajectory analysis, and cell–cell communication analysis, were utilized. B) t‐SNE plot depicting the spatial transcriptomics‐based cell types in PSCC tissues. Each color represents a distinct cell population. C) Expression patterns of key marker genes for each cell type. D) Spatial distributions of B cells, NK and T cells, dendritic cells, and other cell types (including myeloid cells, tumor cells, endothelial cells, and fibroblasts) within PSCC tissues, with TLS regions outlined by dashed circles. E) The PSCC samples were stratified into high‐ and low‐TLS score groups on the basis of the median number of B cells, NK and T cells, and dendritic cells within the TLS regions. The dashed circles indicate the TLS regions, with red boxes representing the high‐TLS score group and blue boxes representing the low‐TLS score group. F) Proportional distributions of B cells, NK and T cells, dendritic cells, and other cell populations across tissues. G) Representative H&E‐stained sections from three samples in the internal cohort, with TLS regions highlighted by yellow circles. Samples with a total TLS area greater than the median (5.16 mm^2^) were classified into the high‐TLS score group, whereas those with a smaller area were classified into the low‐TLS score group. Scale bar = 100 µm. H,I) Kaplan–Meier survival curves comparing the high‐ versus low‐TLS score groups of PSCC patients, revealing that the patients in the low‐TLS score group had a worse prognosis (internal cohort, *n* = 152, *p* < 0.01; external cohort, *n* = 63, *p* < 0.05). Similarly, external cohort samples were classified into high‐ and low‐TLS score groups on the basis of whether the total TLS area exceeded the median (4.76 mm^2^).

This research integrated spatial transcriptomics, scRNA‐seq, and bulk RNA‐seq to systematically investigate the role of TLSs in the immune microenvironment of PSCC, and the findings were validated through multiplex immunofluorescence (mIF). On the basis of these data, we further explored the interactions between B‐cell subsets within TLSs and other immune cells, as well as their potential mechanisms of action (**Figure** **1**A). Considering the critical role of spatial information in accurately characterizing TLS structure, we first performed rigorous batch correction on the spatial transcriptomics data, followed by t‐SNE dimensionality reduction and unsupervised clustering. Based on the high expression of specific marker genes for each cell type, we identified seven major cell populations (Figure 1B; Figure S1A, Supporting Information).These included B cells (*CD79A*, *MS4A1*, and *CD20*), dendritic cells (*ITGAX* and *CD40*), myeloid cells (*CD209*, *CD68*, *CD14*, and *CD163*), NK and T cells (*CD3D*, *CD3E*, and *GNLY*), cancer cells (*CDKN2A*, *EPCAM*, and *KRT18*), endothelial cells (*PECAM1*, *CD34*, and *VWF*), and fibroblasts (*DCN*, *THY1*, and *ACTA2*) (Figure 1C).^[^ ^24^, ^25^ ^]^ The spatial distribution of these cell types is shown in Figure S1B (Supporting Information).

Since TLS formation is driven primarily by the aggregation of immune cells, including B cells, NK and T cells, and dendritic cells, we first localized these populations within the tumor microenvironment by analyzing the in situ expression of specific marker genes for each cell type in TLS regions. We subsequently applied the density‐based clustering algorithm DBSCAN to analyze the aggregation of highly expressed marker genes,^[^ ^26^ ^]^ defining these regions as TLSs (Figure 1D; Figure S1C–E, Supporting Information). To validate the accuracy of this method, we further assessed potential TLS biomarkers, including germinal centers, T follicular helper cells, and chemokine‐related marker genes (e.g., *MKI67, CXCR5*, and *CCL2*)^[^ ^27^ ^]^ (Figure S1F, Supporting Information). These marker genes presented significantly increased expression in TLS regions, confirming the high biological relevance of the TLS regions delineated by DBSCAN. Additionally, the strong correlation between the TLS regions identified by DBSCAN, which were characterized by dense immune marker gene expression, and those confirmed by the intense immunofluorescence signals of CD20, CD3, and CD11c provided strong evidence for the spatial accuracy of the algorithm in identifying biologically relevant TLS structures (Figure S1G, Supporting Information). On the basis of this algorithm, we classified the samples into high‐ and low‐TLS score groups according to the median cell count in the TLS region (Figure 1E). Interestingly, our analysis revealed that across the three immune cell types, B cells represented the predominant immune cell population within TLS regions (Figure 1F). Clinically, we sought to explore whether the presence of TLSs was correlated with improved survival in patients with PSCC. Using the cohort‐specific median cumulative TLS area of 5.16 mm^2^, calculated as the sum of all TLS regions within a single H&E‐stained PSCC tissue section for each of the 152 internal cohort patients, the patients were divided into high‐ and low‐TLS score groups (Figure S1H, Supporting Information). Survival analysis revealed that patients with a high‐TLS density had significantly prolonged OS compared with those with a low‐TLS density (*p* < 0.01, Figure 1G,H). Notably, the same trend was observed in the external cohort (high vs low TLS, *p* < 0.05; Figure 1I).

The presence of TLSs was significantly associated with improved survival in patients with PSCC, and the immune cell composition within TLSs, particularly B cells, may play a crucial role in determining patient prognosis.

### 2.2 CD74⁺ B Cells in TLSs are Key Regulators of the Immune Response and Prognostic Indicators for Improved Survival in PSCC

**Figure 2:**
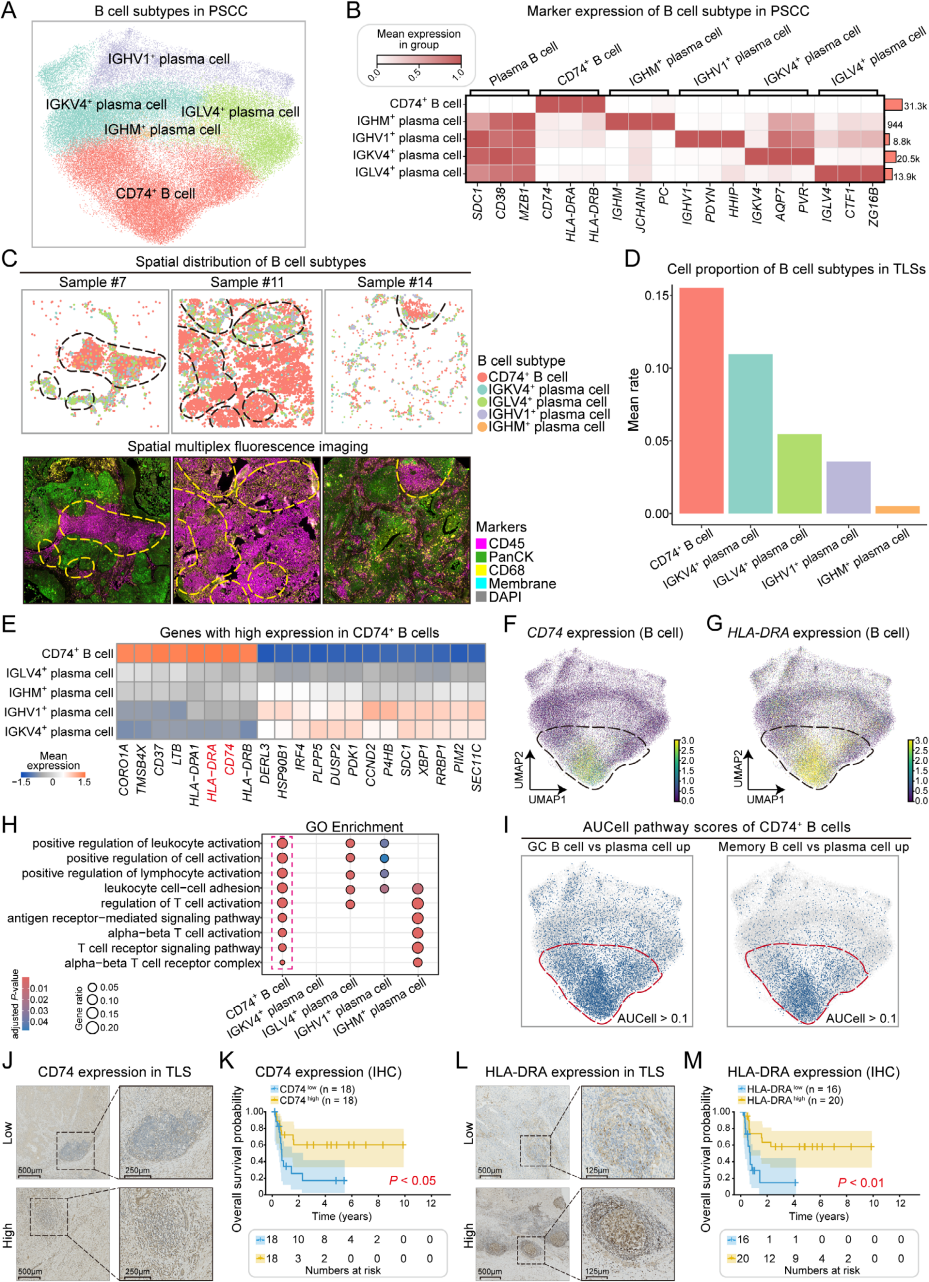
Characterization of B‐cell subtypes from the PSCC spatial transcriptomics data. A) UMAP plot showing the distributions of different B‐cell subtypes. B) Heatmap illustrating the expression levels of markers across B‐cell subtypes. C) Upper panel: spatial distribution of B‐cell subtypes in three representative samples. Lower panel: mIF images of PSCC tissue from the same three representative samples, showing the localization of immune cells (CD45), cancer cells (PanCK), macrophages (CD68), cell membranes, and nuclei (DAPI). Dashed circles indicate TLS regions. D) Proportional representation of B‐cell subpopulations in TLSs compared with all B cells in PSCC tissue. E) Differential gene expression in CD74⁺ B cells, with the highest expression observed for *CD74* and *HLA‐DRA*. The color gradient represents the mean expression levels of the genes. F,G) UMAP plots showing the expression of *CD74* and *HLA‐DRA* in CD74⁺ B cells. H) GO functional enrichment analysis of genes highly expressed in CD74⁺ B cells compared with other subtypes. I) According to the AUCell estimated pathway scores for “GSE12366_GC_BCELL_VS_PLASMA_CELL_UP” (left) and “GSE13411_IGM_MEMORY_BCELL_VS_PLASMA_CELL_UP” (right), CD74⁺ B cells showed transcriptional profiles more closely resembling those of GC B cells and IgM⁺ memory B cells than those of plasma cells. J) Representative IHC images showing the areas of low and high CD74 expression within TLS regions. K) Kaplan–Meier analysis of overall survival in patients with low (*n* = 18) versus high (*n* = 18) CD74 expression in TLSs, *p* < 0.05. L) Representative IHC images showing low and high *HLA‐DRA* expression levels within TLS regions. M) Kaplan–Meier analysis of overall survival in patients with low (*n* = 16) versus high (*n* = 20) *HLA‐DRA* expression in TLSs, *p* < 0.05.

The heterogeneity of B cells was analyzed by identifying five functionally distinct B‐cell subtypes on the basis of the high expression of immunoglobulin (Ig) family genes and antigen‐presentation‐related genes from the spatial transcriptomics data (**Figure** **2**A,B). These subtypes include IGHV1⁺ plasma cells (*IGHV1*, *PDYN*, and *HHIP*), IGKV4⁺ plasma cells (*IGKV4*, *AQP7*, and *PVR*), IGLV4⁺ plasma cells (*IGLV4*, *CTF1*, and *ZG16B*), IGHM⁺ plasma cells (*IGHM*, *JCHAIN*, and *PC*), and CD74⁺ B cells (*CD74*, *HLA‐DRA*, and *HLA‐DRB*).^[^ ^28^, ^29^ ^]^ The first four subtypes are characterized by high expression of distinct Ig heavy or light chain genes, representing diverse plasma cell differentiation states, whereas CD74⁺ B cells predominantly exhibit antigen presentation functions. Based on the spatial transcriptomics analysis, CD74⁺ B cells within TLS regions were found to be spatially colocalized with CD45⁺ immune cells, a pattern that was highly consistent with our mIF observations (Figure 2C; Figure S2A, Supporting Information). Compared with other subtypes, CD74⁺ B cells constituted the highest proportion of B cells within the TLS (Figure 2D). To further investigate the functional characteristics of CD74⁺ B cells, we observed that key markers, such as *CD74* and *HLA‐DRA*, were significantly upregulated in this subset (Figure 2E–G). Notably, bulk RNA‐seq further revealed that elevated expression levels of these genes were significantly associated with prolonged survival in patients with PSCC (*p* < 0.05; Figure S2B,C, Supporting Information). In addition, gene pathway enrichment analysis highlighted the pivotal role of CD74⁺ B cells in immune responses, particularly in biological processes related to leukocyte activation, lymphocyte activation, and T‐cell regulation (Figure 2H). Furthermore, compared with plasma cells, CD74⁺ B cells presented higher pathway activity scores in germinal‐center‐B‐cell‐ and memory‐B‐cell‐related pathways, suggesting that their immune response function aligned more closely with the characteristics of germinal center B cells and memory B cells (Figure 2I).

Given the critical involvement of CD74 and *HLA‐DRA* in immune regulation, we assessed whether their expression levels within TLS regions could serve as prognostic indicators. By stratifying patients with PSCC into high‐ and low‐expression groups on the basis of CD74 and *HLA‐DRA* expression within TLSs, we found that higher levels of both genes were significantly correlated with improved overall survival (*CD74*: *p* < 0.05, Figure 2J,K; and *HLA‐DRA*: *p* < 0.01, Figure 2L,M), suggesting their potential role in mediating TLS‐associated antitumor immune responses.

Furthermore, in a comparative analysis of different B cell subtypes using both AUCell and CIBERSORT methods, we found that only CD74⁺ B cells showed significantly higher signature scores in patients, and this was strongly and positively correlated with better clinical outcomes, whereas no significant differences were observed in other plasma cell subtypes (Figure S2D–L, Supporting Information). These findings collectively indicated that CD74⁺ B cells not only dominated the TLS microenvironment but also played a critical role in immune modulation. As such, their elevated signature score may serve as a promising prognostic biomarker indicative of survival benefit in patients with PSCC.

### 2.3 CD74⁺ B Cells in PSCC TLSs are Essential for Immune Activation and Early Differentiation

**Figure 3:**
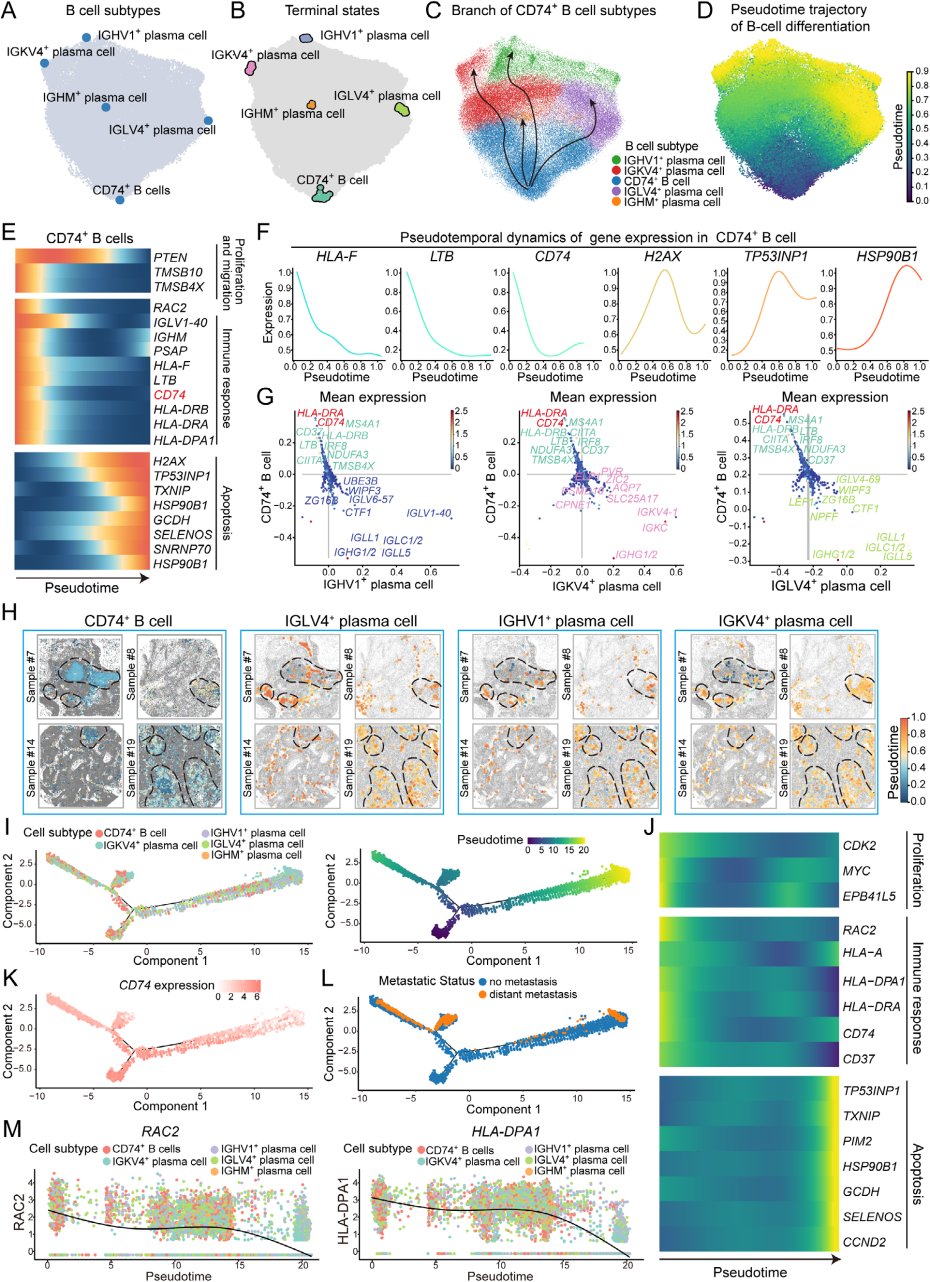
Pseudotime analysis of CD74⁺ B cells in PSCC tissue on the basis of the spatial transcriptomics and scRNA‐seq data. The analyses in (A–H) were based on spatial transcriptomic data from PSCC samples, and those in (I–M) were based on scRNA‐seq data from PSCC samples. A,B) UMAP plots showing the spatial distribution and pseudotime endpoints of B‐cell subtypes. C) Pseudotime developmental trajectory of CD74⁺ B cells. D) UMAP visualization of B‐cell development, with yellow tones indicating later stages of cell development. E) Pseudotime analysis revealed that the expression genes related to proliferation, migration, and the immune response in CD74⁺ B cells decreased over time, whereas genes related to apoptosis showed the opposite trend, with redder colors indicating higher gene expression. F) Pseudotime expression dynamics of *HLA‐F*, *LTB*, *CD74*, *H2AX*, *TP53INP1*, and *HSP90B1* in CD74⁺ B cells. G) Key regulatory genes involved in the development of CD74⁺ B cells, with dots color‐coded according to gene expression level; red indicates increased expression. H) Pseudotime developmental evolution of B‐cell subtypes across patient samples, with colors transitioning from light blue to orange–red indicating progression from the early to late developmental stages. The dashed circles indicate TLS regions. I) Pseudotime analysis of the development of five distinct B‐cell subtypes, with colored dots representing different cell subtypes. J) Pseudotime analysis revealed that the expression of genes associated with proliferation and the immune response in CD74⁺ B cells decreased over time, whereas the expression of genes related to proliferation increased, with yellow indicating increased expression levels. K) The expression of *CD74* gradually decreased during development, with lighter colors representing lower expression levels. L) Pseudotime analysis based on patient metastasis status, with blue dots representing M0 (no distant metastases) and orange dots representing M1 (distant metastasis). M) Pseudotime expression dynamics of the immune response‐related gene *RAC2* and the antigen‐presentation‐related gene *HLA‐DPA1* in B‐cell subtypes, with the expression of *HLA‐DPA1* gradually decreasing over time in CD74⁺ B cells (orange dots).

Considering the dynamic nature of TLS formation, the known differentiation of plasma cells from mature B cells upon antigen stimulation within TLSs, and the role of *CD74* in facilitating B‐cell differentiation,^[^ ^16^ ^]^ we based our analysis on Palantir, where CD74⁺ B cells were set as the starting point of development, and other subtypes were set as endpoints to map the in situ developmental trajectory of B cells within TLSs (**Figure** **3**A–D). During the development of CD74⁺ B cells, genes associated with cell proliferation and migration, such as *PTEN* and *TMSB10*, exhibited gradual downregulation, while apoptosis‐related genes showed an increase in expression in the later stages of development, which was consistent with the characteristic features of CD74⁺ B cells as a developmental starting point. Moreover, genes involved in immune responses were upregulated during the early stages of development, exhibiting a subsequent decrease in expression with development progression, consistent with our findings of immune response pathway enrichment in CD74⁺ B cells (Figure 3E,F; Figure S3A, Supporting Information). Analysis of genes driving the differentiation of CD74⁺ B cells into various plasma cell subtypes showed consistently elevated levels of *CD74* and *HLA‐DRA*, whereas these genes were not expressed during plasma cell differentiation and thus not key drivers (Figure 3G; Figure S3B, Supporting Information). Interestingly, we observed that CD74⁺ B cells in the TLSs of patients with PSCC predominantly remained in the early developmental stages, whereas plasma cells exhibited a disordered and irregular developmental pattern and lacked a clear trajectory (Figure 3H; Figure S3C, Supporting Information). These findings suggested that CD74⁺ B cells, which are predominant in early development, exhibited stronger proimmune response functions compared with plasma cells.

Furthermore, to validate these findings, we sorted CD74^−^ B cells and CD74⁺ B cells from tissue samples of six PSCC patients and cultured them in vitro (Figure S3D, Supporting Information). At days 0, 3, 7, and 10 of culture, we assessed the expression of plasma cell markers CD138, CD38, Blimp‐1, and XBP1 to evaluate and compare the role of CD74 in driving B cell differentiation into plasma cells. The results showed that the plasma cell markers of CD74⁺ B cells gradually upregulated over time, displaying stronger differentiation potential, and their differentiation ability was significantly superior to that of CD74^−^ B cells (Figure S3E, Supporting Information). These experimental results further support our hypothesis that CD74⁺ B cells are key precursor cells driving plasma cell differentiation and immune responses.

Moreover, the scRNA‐seq data corroborated these findings. We first performed integrative analysis on batch‐corrected scRNA‐seq datasets and annotated cell populations on the basis of established lineage‐specific marker genes. This approach enabled the high‐resolution classification of heterogeneous cellular components within the tumor microenvironment, ultimately delineating seven major cell clusters, including NK and T cells, B cells, macrophages, mast cells, fibroblasts, endothelial cells, and epithelial cells (Figure S3F–H, Supporting Information). Among these, B cells were classified into five distinct subtypes. The pseudotime analysis results revealed that CD74⁺ B cells occupied the initial positions on the developmental trajectory, indicating their role as progenitors of other B‐cell subtypes (Figure 3I). During development, the expression of genes related to proliferation, migration, and immune responses decreased, whereas the expression of apoptosis‐associated genes increased (Figure 3J). Additionally, *CD74* expression was high in the early stages of development and gradually decreased as development progressed (Figure 3K). Pseudotime analysis of patients with metastatic and nonmetastatic disease further revealed that CD74⁺ B cells in patients with distant metastasis presented significantly delayed development, suggesting a potential link between the development or function of CD74⁺ B cells and metastasis (Figure 3L). Finally, among the CD74⁺ B‐cell subtypes, the expression of genes related to proliferation, migration, and immune responses, such as *RAC2*, *HLA‐DPA1*, *CORO1A*, *CD73*, *CD74*, and *HLA‐B*, decreased during development, whereas the expression of apoptosis‐related genes, including *CYBA*, *HSP90B1*, and *SELENOS*, increased (Figure 3M; Figure S3I,J, Supporting Information). Notably, *CD74* and other antigen‐presentation‐related genes reemerged, with their expression patterns aligning with the trends observed in our spatial transcriptomics analysis.

### 2.4 CD74⁺ B Cells in TLSs Display Markedly Enhanced Immune Functions Compared with Those in Non‐TLSs (nTLSs)

**Figure 4:**
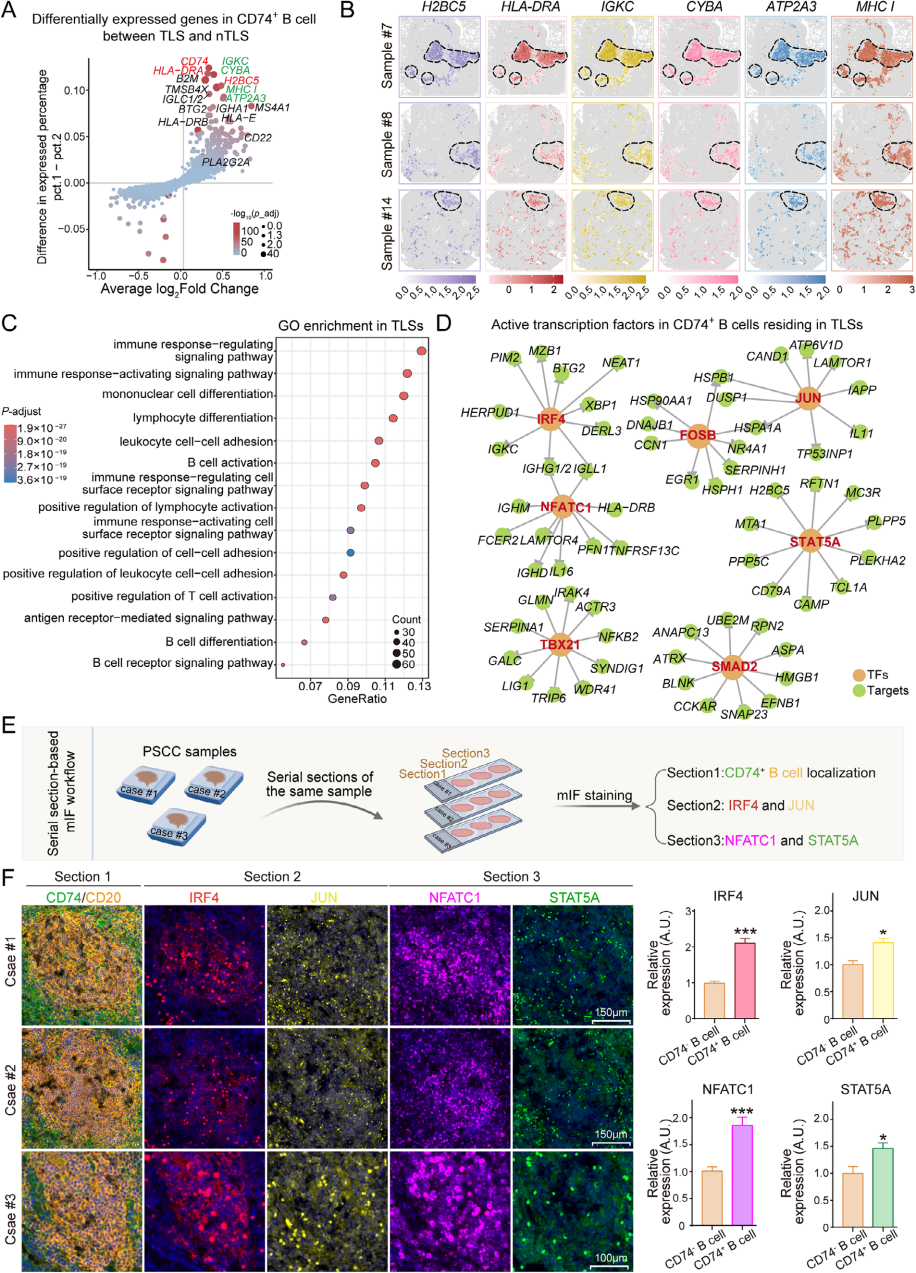
Gene expression in and functional analysis of CD74⁺ B cells within and outside TLSs in PSCC tissues. A) Volcano plot of the differentially expressed genes in *CD74⁺* B cells from TLSs compared with those from nTLSs, with larger and redder dots indicating higher expression levels. B) Spatial in situ expression of *H2BC5*, *HLA‐DRA*, *IGKC*, *CYBA*, *ATP2A3*, and *MS4A1*, with deeper colors representing higher expression levels; dashed circles denote TLS regions. C) Bubble plot showing the results of the GO enrichment analysis of genes upregulated in *CD74⁺* B cells within TLSs compared with those in nTLS regions. D) Transcription factors exhibiting greater activity in *CD74⁺* B cells from TLSs than in B cells from nTLSs. E) Workflow of the use of serial tissue sections from the same PSCC patient sample. The first section was used to localize CD74⁺ B cells within TLS regions, whereas the remaining two sections were used to assess the expression of key transcription factors, such as *IRF4*, *JUN*, *NFATC1*, and *STAT5A*, within the same TLS‐localized CD74⁺ B‐cell populations. F) Representative image of mIF staining of PSCC tissue sections from three patients. Left panel: Section 1 was stained with CD74 (green) and CD20 (orange) to localize CD74⁺ B cells within TLS regions. Sections 2 and 3 show the expression of *IRF4* (red), *JUN* (yellow), *NFATC1* (purple), and *STAT5A* (green) at the corresponding TLS locations. Right panel: quantification of transcription factor expression levels in CD74^−^ B cells and CD74⁺ B cells. Two‐sided Wilcoxon test, *p < 0.05, ***p < 0.001.

The significant early immune activity exhibited by CD74⁺ B cells within TLSs prompted an interesting question about whether this activity was driven by their unique aggregation within the TLS regions or by the intrinsic functional characteristics of these cells. To explore this hypothesis, we compared the functional differences between CD74⁺ B cells in the TLS and nTLS regions. Differential gene expression analysis, visualized with a volcano plot, revealed that genes such as *H2BC5*, *HLA‐DRA*, *IGKC*, *CYBA*, *ATP2A3*, and *MHCI* class were significantly upregulated in CD74⁺ B cells within TLSs, which aligned with the high enrichment of these cells in the TLS regions (**Figure** **4**A,B). Specifically, *H2BC5* contributed to the stability of gene expression in B cells, while high expression of *HLA‐DRA* ^[^ ^30^ ^]^ facilitates antigen presentation by B cells to T cells, thus stimulating immune responses and enhancing B‐cell immune surveillance.^[^ ^30^, ^31^, ^32^ ^]^ Moreover, *IGKC* plays a crucial role in enhancing the antibody production capacity of B cells, whereas *CYBA* is essential for providing an energy supply, ensuring proper cellular function.^[^ ^33^, ^34^ ^]^ *ATP2A3*, which regulates the calcium ion concentration, is indispensable for B‐cell signaling and activation.^[^ ^35^ ^]^ The coordinated expression of these genes promotes B‐cell recognition, activation, and antibody production, thereby playing a pivotal role in adaptive immune responses. Notably, these highly expressed genes are involved primarily in immune regulation, immune response activation, and lymphocyte differentiation, with several pathways closely linked to T‐cell activation (Figure 4C).

Previous studies have shown that the maturity of TLS is closely related to B‐cell function. Therefore, we classified the maturity of TLS using a single sample gene set enrichment analysis (ssGSEA) scoring method based on germinal center markers, including *BCL6*, *AICDA*, *CD38*, *ICOS*, *CXCR5*, and *CXCL13* (Figure S4A, Supporting Information). TLSs with a score greater than or equal to the cohort median were defined as mature, while those with a score lower than the median were considered immature (Figure S4B, Supporting Information). We then performed differential gene expression analysis of B cells from mature and immature TLSs. The results showed that six genes related to plasma cell function were significantly upregulated in B cells from mature TLSs, including *IGLV6‐57*, *IGHG1/2*, *IGKC*, *MZB1*, *CD38*, and *TNFRSF17* (Figure S4C, Supporting Information). The high expression of these genes suggests that B cells in mature TLSs may have entered the plasma cell differentiation process. To further explore the functions of these highly expressed genes, we performed pathway enrichment analysis. The results showed that in B cells from mature TLSs, 10 key immune‐related pathways were activated, including the B‐cell receptor signaling pathway, adaptive immune response, response to endoplasmic reticulum stress, antigen‐receptor‐mediated signaling pathway, and immune effector processes (Figure S4D, Supporting Information). These findings suggest that mature TLSs, by providing a microenvironment that supports plasma cell differentiation and antibody secretion, further amplify the early immune activation observed in their CD74⁺ B cells, driving an efficient adaptive immune response, with both processes working in concert.

Compared with CD74⁺ B cells in nTLS regions, those in TLS regions, including *STAT5A*, *IRF4*, *NFATC1*, *JUNB*, *FOSB*, and *TBX21*, exhibited markedly greater transcription factor activity (Figure 4D). The activation of these transcription factors not only aids in the proliferation and differentiation of and antibody production by B cells but also enhances their ability to recognize and respond to antigens.^[^ ^36^, ^37^, ^38^ ^]^ Furthermore, given the critical role of B cells in antigen presentation, the activation of these transcription factors indirectly promotes T‐cell activation and proliferation, thereby strengthening T‐cell responses to pathogens.^[^ ^39^, ^40^ ^]^

Consequently, we collected three consecutive tissue sections from each patient. The first section was employed to precisely identify CD74⁺ B cells within TLS regions, whereas the subsequent two sections were utilized to assess the expression of key transcription factors—namely, *IRF4*, *JUN*, *NFATC1*, and *STAT5A*—within the same localized CD74⁺ B‐cell populations (Figure 4E). Notably, the expression of these transcription factors was significantly elevated in CD74⁺ B cells residing in TLSs, with particularly pronounced upregulation of *IRF4* and *NFATC1* (Figure 4F). The activation of these transcription factors endowed TLS‐resident CD74⁺ B cells with enhanced immunological competence, highlighting their potential as prognostic biomarkers and therapeutic targets in cancer immunotherapy.

### 2.5 NK and T Cells are the Primary Targets of CD74⁺ B Cells within TLSs

**Figure 5:**
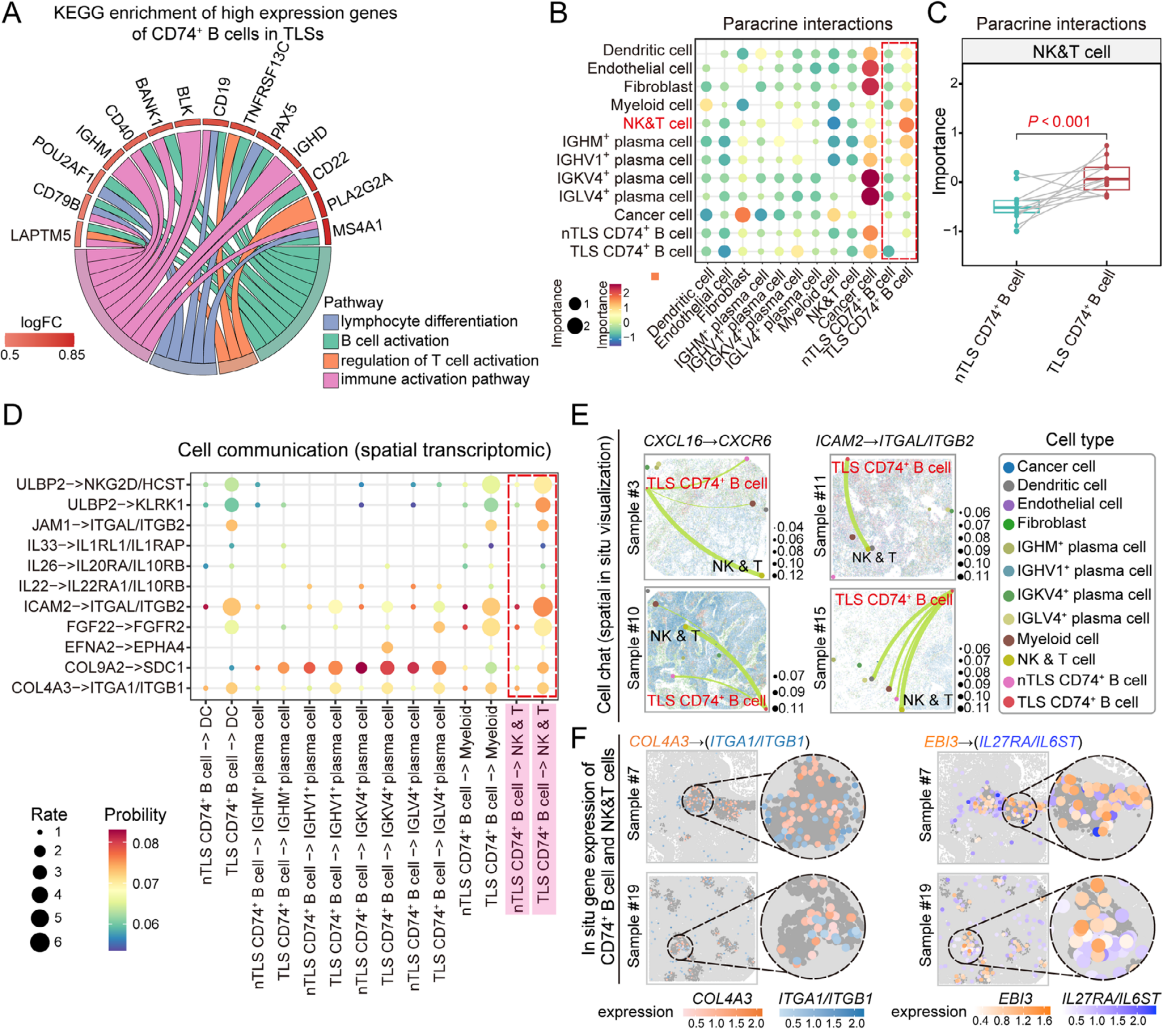
Communication of CD74⁺ B cells with other cell types in TLSs and nTLSs. A) KEGG enrichment analysis of highly expressed genes in *CD74⁺* B cells within TLS regions. B) Dot plot of cell–cell interactions, with signaling cells on the *x*‐axis and target cell types on the *y*‐axis. The dot size represents the relative importance of each interaction, and the color indicates the interaction strength from low (blue) to high (red). C) Comparison of signaling interactions between *CD74⁺* B cells and NK and T cells in the TLS and nTLS regions. Two‐sided Welch *t*‐test (unpaired), *n* = 18, *p* < 0.001. D) Dot plot illustrating communication between *CD74⁺* B cells and other cells in the TLS and nTLS regions. Larger dots indicate a higher probability of communication, and redder colors denote a stronger probability of interaction. E) Spatial mapping of cell–cell communication on the basis of key ligand–receptor pairs, where line thickness indicates interaction strength, and different cell types are represented by colored dots. F) Spatial coexpression of key ligand–receptor pairs between *CD74⁺* B cells and NK and T cells. The gray areas indicate TLS regions, and deeper colors correspond to higher gene expression levels.

Next, we explored how the aggregative characteristics of CD74⁺ B cells within TLSs bolstered their robust immune response. KEGG pathway analysis revealed that, compared with CD74⁺ B cells in nTLS regions, those within TLSs presented increased expression of genes associated with four principal pathways; among them, the pathway governing the modulation of self‐immune responses stood out, alongside a distinctive pathway regulating T‐cell activation (**Figure** **5**A). Notably, cell interaction analysis revealed that CD74⁺ B cells within TLSs had significantly stronger interactions with other immune cells than their counterparts in nTLS regions did; in particular, their interaction with NK and T cells was more pronounced (Figure 5B,C; Figure S5, Supporting Information). An in‐depth analysis of cellular communication demonstrated that CD74⁺ B cells in TLSs exhibited markedly enhanced communication with NK and T cells via the influence of multiple key ligand–receptor pairs, further suggesting that NK and T cells were the primary targets of CD74⁺ B cells within TLSs (Figure 5D; Figure S6A, Supporting Information). For example, ligand–receptor pairs such as CXCL16–CXCR6 and ICAM–ITGAL/ITGB2 facilitated cell migration, whereas RLN3–RXFP4 and CTSG–F2RL3 regulated the proliferation, differentiation, and activation of immune cells, propelling the immune response forward (Figure 5E; Figure S6B, Supporting Information). The spatial colocalization of these ligand–receptor pairs within TLSs further substantiated their intimate associations within immune cells (Figure 5F; Figure S6C,D, Supporting Information).

In summary, CD74⁺ B cells within TLSs, through their intricate interactions with NK and T cells, likely played indispensable roles in promoting their proliferation, migration, and activation, thereby occupying a pivotal position within the tumor immune microenvironment.

### 2.6 The Naive T‐Cell Subset within TLSs has a Heightened Ability to Mount an Immune Response

**Figure 6:**
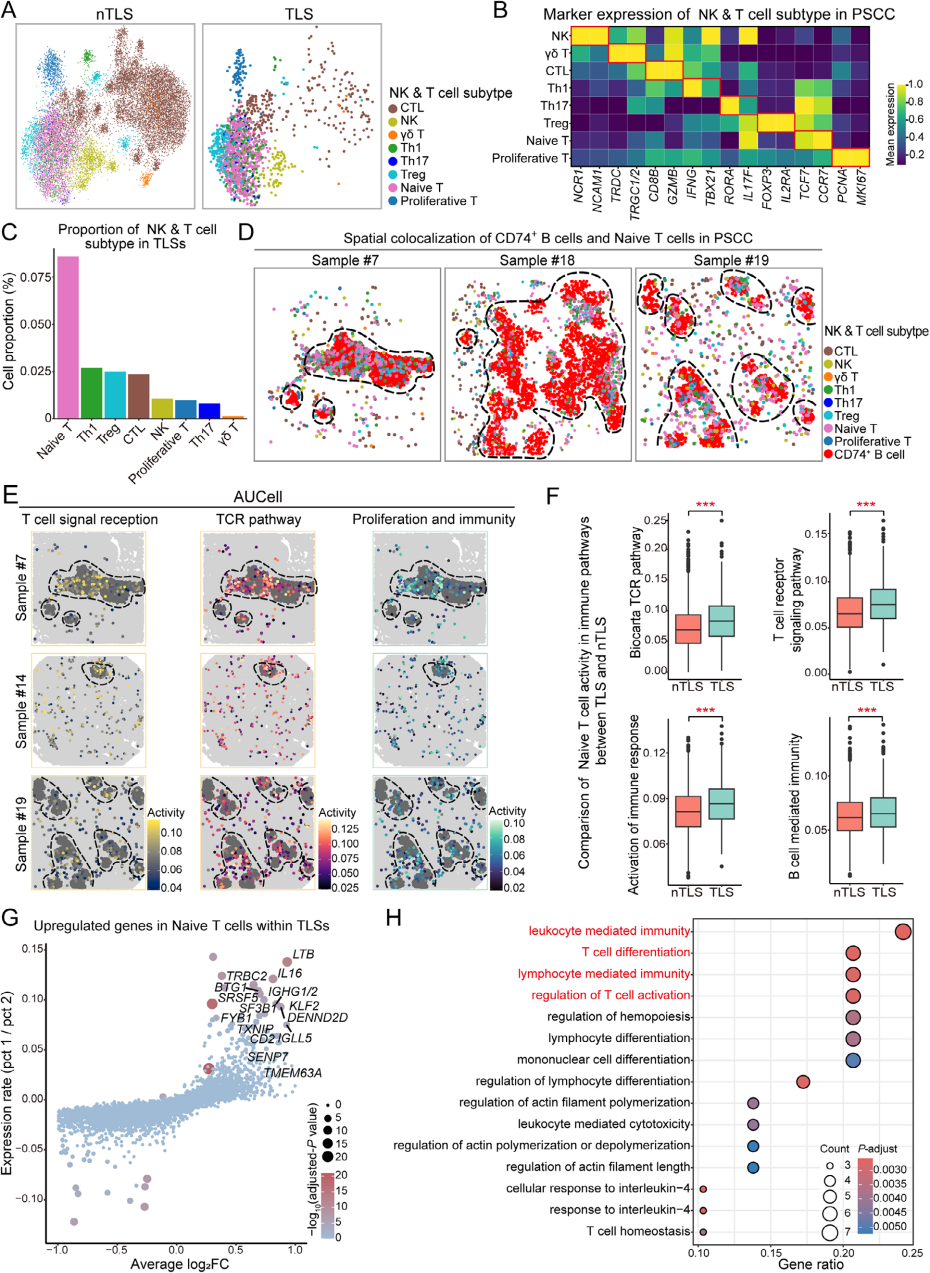
Features and functional differences between naive T cells in TLSs and nTLSs. A) UMAP plot showing the distribution of NK‐ and T‐cell subtypes in nTLSs and TLSs. B) Expression of marker genes of each cell subtype, including NK cells (*NCR1* and *NCAM1*), γδ T cells (*TRDC* and *TRGC1/2*), CTLs (*CD8B* and *GZMB*), Th1 cells (*IFNG* and *TBX21*), Th17 cells (*RORA* and *IL17F*), Tregs (*FOXP3* and *IL2RA*), naive T cells (*TCF7* and *CCR7*), and proliferating T cells (*PCNA* and *MKI67*). C) Average proportions of each cell subtype within TLSs. D) Spatial localization of each cell type in representative patient samples. The dashed circles indicate TLS regions. E) Representative AUCell analysis of naive T‐cell pathway activity based on MSigDB‐curated gene sets. Lighter colors indicate higher activity, and gray areas represent TLS regions. F) Comparison of naive T‐cell pathway activity between the nTLS and TLS regions on the basis of MSigDB. G) Volcano plot showing differential gene expression in naive T cells from TLSs compared with those from nTLS regions. H) GO enrichment analysis of genes upregulated in naive T cells from TLSs compared with those from nTLSs. The dot size indicates the number of genes, and the color scale represents statistical significance. ***, *p* < 0.001.

Building upon the identification of NK and T cells as the primary targets of CD74⁺ B cells within TLSs, we proceeded to pinpoint more specific subpopulations of these immune cell classes that are influenced by CD74⁺ B cells. From the null transcriptome data, we identified eight subpopulations of NK and T cells on the basis of the high expression of their classic biomarkers and divided these cells into TLS and nTLS groups (**Figure** **6**A,B). The NK and T cell subtypes include NK cells (*NCR1*, *NCAM1*), γδ T cells (*TRDC*, *TRGC1/2*), CTLs (*CD8B*, *GZMB*), Th1 cells (*IFNG*, *TBX21*), Th17 cells (*RORγA*, *IL17F*), Treg cells (*FOXP3*, *IL2RA*), naive T cells (*TCF7*, *CCR7*), and proliferative T cells (*PCNA*, *MKI67*).^[^ ^41^, ^42^ ^]^ Within TLSs, the naive T‐cell subpopulation was notably predominant and these cells were primarily concentrated in TLS regions, exhibiting significant spatial colocalization with CD74⁺ B cells (Figure 6C,D; Figure S7A, Supporting Information). Functionally, naive T cells in TLSs presented evident activation of pathways associated with T‐cell signaling, TCRs, and immune response regulation, particularly proliferation and activation (Figure 6E; Figure S7B, Supporting Information). Pathway activation, quantified by AUCell using MSigDB hallmark gene sets, also revealed that naive T cells within TLSs were more responsive to TCRs, immune response activation, T‐cell receptor signaling, and B‐cell regulation pathways of the immune response (Figure 6F). Our analysis of gene expression profiles between naive T cells in the TLS and nTLS regions revealed that genes such as *LTB*, *IL16*, I*GHG1/2*, and *CD2* were significantly upregulated in naive T cells in the TLS region. These genes are vital for the early development, activation, and functional stability of T cells (Figure 6G). Enrichment analysis of these upregulated genes revealed that naive T cells in TLSs were involved primarily in immune regulation, T‐cell differentiation, and pathway activation (Figure 6H).

Overall, naive T cells in TLSs displayed distinctive functional benefits over those in nTLSs, particularly regarding immune signaling and regulatory activity, a response that may be regulated by CD74⁺ B cells in TLSs.

### 2.7 CD74⁺ B Cells Enhance the Immune Response by Interacting with CD4 on Naive T Cells in TLSs via *HLA‐DRA*

**Figure 7:**
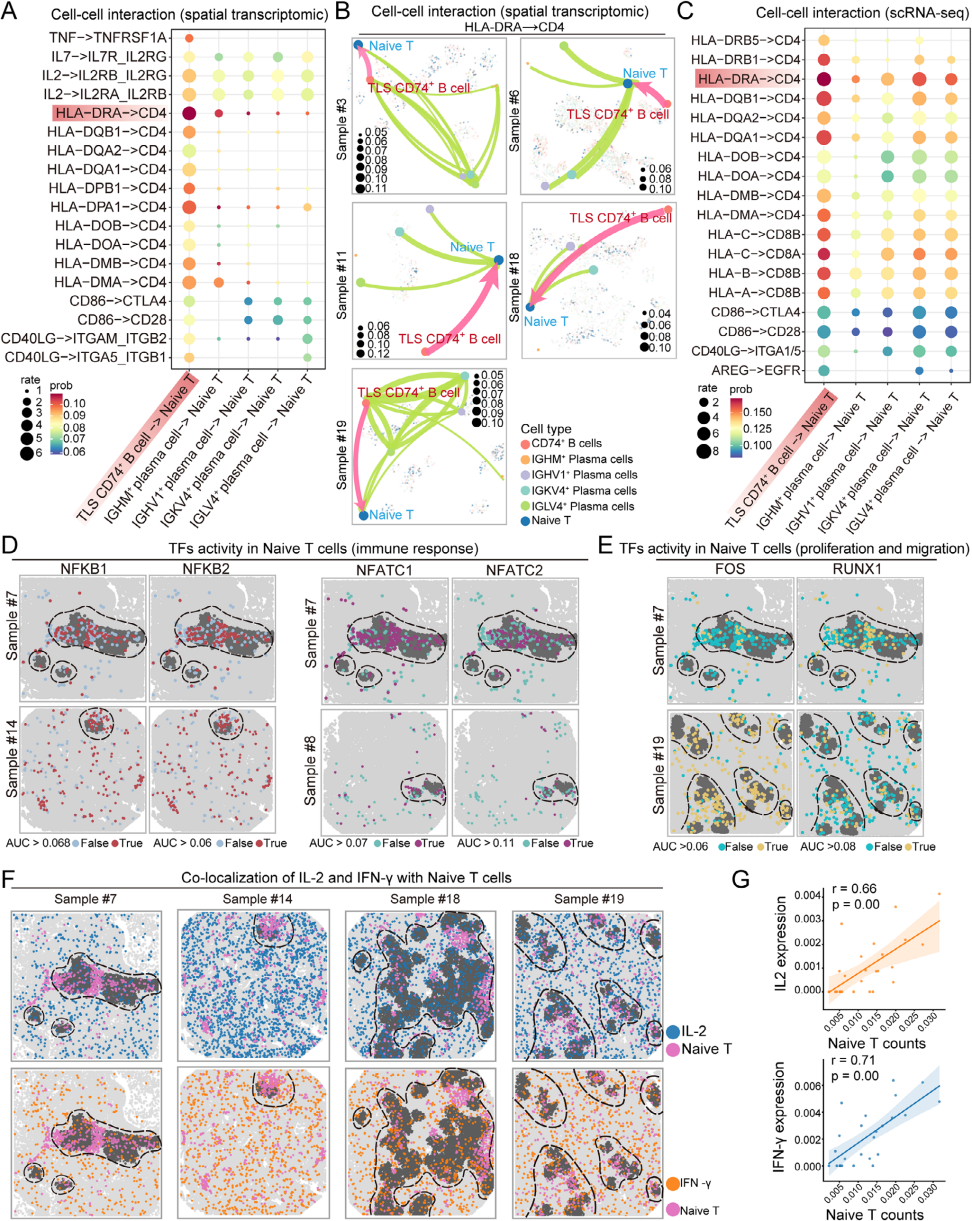
Naive T‐cell communication and activation. A) Cell–cell interaction analysis based on the spatial transcriptomic data. The dot size indicates the interaction probability, and redder colors represent a greater likelihood of interaction. B) Spatial in situ visualization of key ligand–receptor interactions. C) Cell–cell interaction analysis based on the scRNA‐seq data. The dot size indicates the interaction probability, and redder colors represent a greater likelihood of interaction. D,E) In situ expression of transcription factors related to immunity, proliferation, and migration in naive T cells. “True” indicates transcription factor activation, whereas “false” indicates inactivity. The dashed boxes mark the TLS regions. F) Spatial colocalization of naive T cells with *IL2* and *IFNG*. The dashed circles indicate TLS regions. G) Scatter plot showing the correlation between the number of naive T cells and the expression levels of *IL‐2* and *IFN‐γ*.

The above observations prompted us to investigate the cellular interactions responsible for such activation, with a focus on the crosstalk between naive T cells and CD74⁺ B cells within TLSs. By integrating the spatial transcriptomics and single‐cell data, we conducted an in‐depth analysis of the communication between CD74⁺ B cells and other B‐cell subtypes with naive T cells. As anticipated, compared with other B‐cell subtypes, CD74⁺ B cells within TLSs demonstrated the most robust interaction with naive T cells (**Figure** **7**A). While some ligand–receptor pairs were associated with proliferation and migration, the most striking interactions were predominantly related to MHC class I antigen presentation, with the most intense communication observed between *HLA‐DRA* on CD74⁺ B cells and CD4 on naive T cells within TLSs (Figure 7B). This pattern of cellular communication was highly consistent with the scRNA‐seq data from patients with PSCC (Figure 7C). B cells interact with T cells via MHC II molecules and CD4 receptors, a central mechanism in adaptive immune responses that can drive T‐cell functionality. Indeed, we observed the significant upregulation of transcription factors associated with T‐cell activation and immune responses, including *NFKB1*, *NFKB2*, *NFATC1*, and *NFATC2*, in naive T cells (Figure 7D; Figure S8A, Supporting Information). Additionally, transcription factors related to proliferation and migration, such as *FOS* and *RUNX1*, also exhibited marked activation (Figure 7E; Figure S8B, Supporting Information). Furthermore, the increased expression of *IFN‐γ* and *IL‐2* in naive T cells typically signifies effective antigen presentation and costimulation; these cells are no longer in a quiescent state but are undergoing initial activation and differentiation into effector T cells (Figure 7F). The colocalization of these two genes with naive T cells was positively correlated (Figure 7G).

### 2.8 CD74⁺ B Cells as Potential Immunotherapeutic Targets in PSCC

**Figure 8:**
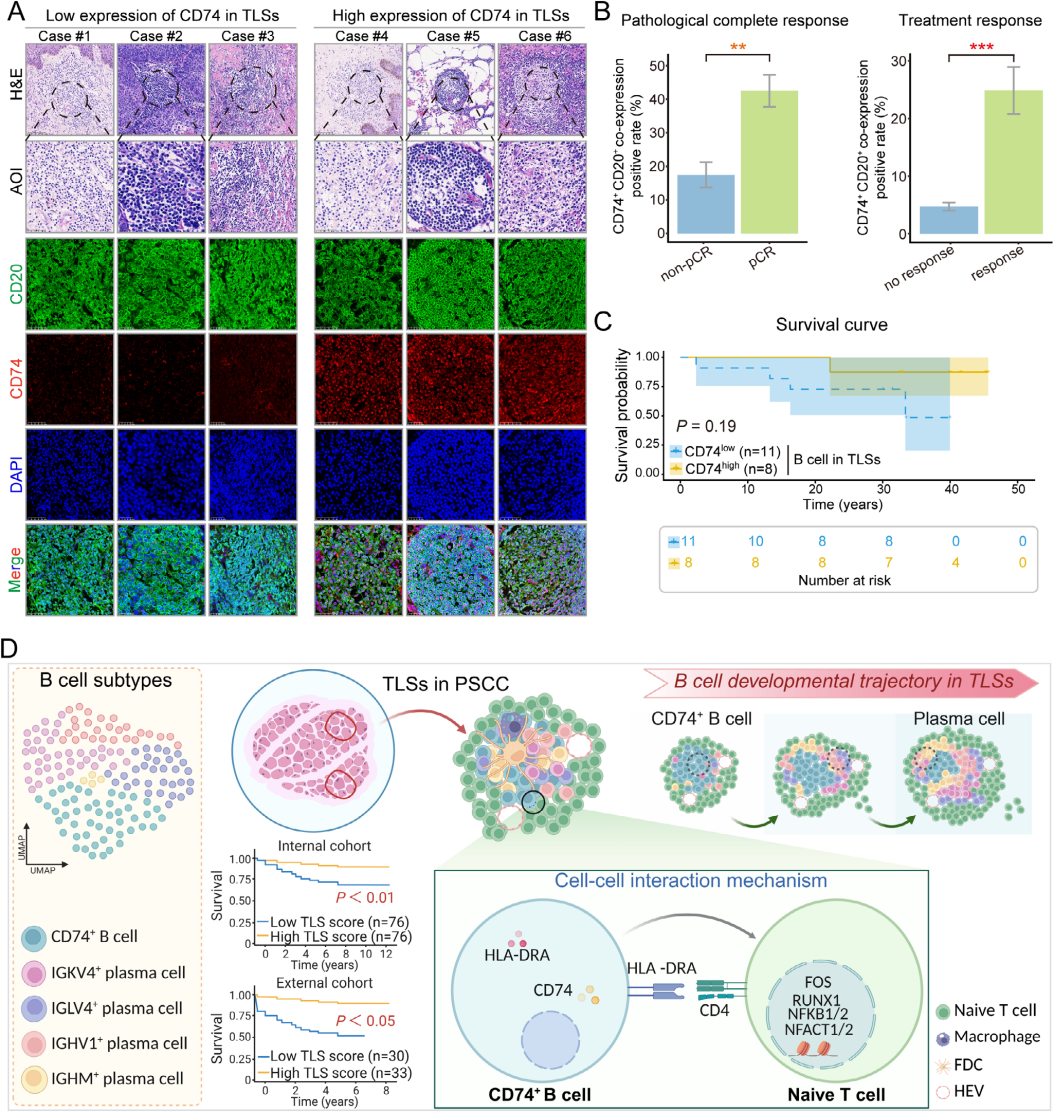
A high proportion of CD74⁺ B cells within TLSs is correlated with improved clinical prognosis in PSCC patients. A) Representative images of immunofluorescence staining for CD20 (green), CD74 (red), and DAPI (blue) in PSCC tissue sections. The area of interest (AOI) is outlined by a dashed line, scale bar = 50 µm. B) Quantitative analysis of CD74⁺CD20⁺ B‐cell coexpression in all enrolled patients (*n* = 29), stratified by pathological complete response (pCR; left) and radiological treatment response (right). pCR, pathological complete response, wherein the response evaluation criteria followed RECIST 1.1; CR, complete response; PR, partial response; SD, stable disease; PD, progressive disease; RECIST, response evaluation criteria in solid tumors. C) Kaplan–Meier survival curve analysis of patients with PSCC with high (*n* = 8) or low (*n* = 11) CD74 expression in B cells in TLSs; *p* = 0.19. D) Differentiation and immune activation response patterns of CD74⁺ B cells in the TLSs of PSCC patients. Survival analysis revealed that patients with higher TLS scores had better prognoses. The differentiation of CD74⁺ B cells into various plasma cell subgroups, including IGKV4⁺, IGLV4⁺, IGHV1⁺, and IGHM⁺ plasma cells, was considered the starting point of the developmental trajectories. In the TLSs of PSCC tissues, CD74⁺ B cells activate immune responses, proliferation, and migration‐related transcription factors in naive T cells via the *HLA‐DRA* ligand and CD4 receptor, promoting immune activation within the tumor microenvironment. ** *p* < 0.01 and *** *p* < 0.001.

Evidence from integrated transcriptomic profiling indicated that a high abundance of CD74⁺ B cells within TLSs was positively associated with improved patient survival and functioned as a critical initiator of naive T‐cell activation, effectively serving as a molecular switch initiating antitumor immune responses. This finding prompted us to further investigate the potential of CD74⁺ B cells as therapeutic targets in clinical settings. Thus, we employed mIF staning to evaluate CD74 expression within TLSs and used CD20 as a canonical B‐cell marker to assess the spatial distribution and density of CD74⁺ B cells. The results revealed markedly increased coexpression of CD74 and CD20 within TLS regions, highlighting the central position of CD74⁺ B cells in the tumor immune microenvironment (**Figure** **8**A). Furthermore, we analyzed data from 29 patients enrolled in a single‐arm phase II clinical trial conducted at our center. All patients received the TNT regimen, which consisted of toripalimab (a PD‐1 inhibitor), nimotuzumab (an EGFR‐targeting monoclonal antibody), and taxane‐based chemotherapy. The results demonstrated that the percentage of CD74⁺CD20⁺ B cells was significantly higher in patients who achieved a pathological complete response (pCR) than in those who did not. In addition, patients classified as responders on the basis of radiological and pathological assessments indicating complete or partial response (CR or PR) also exhibited markedly higher CD74 and CD20 coexpression than nonresponders with stable or progressive disease (SD or PD) (Figure 8B).

These findings suggested a strong association between CD74⁺ B‐cell enrichment and favorable treatment responses. Although Kaplan–Meier survival analysis did not reveal a statistically significant difference between patients with high and low CD74 expression, a clear trend toward better survival outcomes was observed in the high‐expression group (Figure 8C).

Taken together, our findings established a robust link between the presence of TLSs and enhanced survival in patients with PSCC, confirming the critical role of TLSs in shaping the tumor immune microenvironment. We identified a subset of CD74⁺ B cells within TLSs that displayed early developmental plasticity and the potential for plasma cell differentiation. These cells exhibited elevated expression of *HLA‐DRA* and *CD74* and engage in antigen presentation through interaction with CD4 receptors on naive T cells, thereby amplifying immune activation. Specifically, this interaction promotes the upregulation of key transcription factors associated with proliferation and immune activation in naive T cells, increasing their proliferative capacity, migratory potential, and effector functions (Figure 8D). Additionally, the increased expression of *IL‐2* and *IFN‐γ* further supported the activation of naive T cells and the initiation of adaptive immune responses. Collectively, these results suggested that CD74⁺ B cells might serve as promising therapeutic targets in PSCC and may contribute to improved clinical outcomes.

## 3 Discussion

In this study, we provide the first integrated transcriptomic‐based evidence that a high abundance of TLSs and their enriched CD74⁺ B‐cell subset are closely associated with an improved prognosis in patients with PSCC. Notably, CD74⁺ B cells within TLSs are pivotal players in immunoregulation that critically shape the tumor immune landscape. This subset not only exhibits early developmental plasticity and the potential to differentiate into plasma cells but also mediates antigen presentation through the interaction between *HLA‐DRA* and CD4 receptors on naive T cells, thereby triggering robust T‐cell immune activation and enhancing the overall immune response. In summary, this study offers a novel perspective on the spatial and functional identity of CD74⁺ B cells within the PSCC tumor immune microenvironment (Figure 8D). These findings not only broaden our understanding of the antitumor mechanisms of TLSs but also highlight CD74⁺ B cells as promising therapeutic targets for the development of more precise immunotherapeutic strategies for PSCC.

Our initial findings underscore a positive correlation between the presence of tumor‐associated TLSs and favorable patient outcomes in patients with PSCC, a trend observed consistently across both internal and external patient cohorts. More notably, we identified a specific B‐cell subset, CD74⁺ B cells, the presence and gene expression levels of which were also positively associated with an improved prognosis in PSCC patients. This observation is particularly important as it contrasts with findings in other cancers, such as in cervical cancer where CD74⁺ macrophages induce M2 macrophage polarization to promote immunosuppression, or in triple‐negative breast cancer where CD74 on DCs promotes an immunosuppressive microenvironment by inducing the expansion of tolerant dendritic cells and regulatory B cells.^[^ ^43^, ^44^ ^]^ This discrepancy likely stems from the highly context‐dependent nature of CD74's function, which is influenced by both cell type and the tumor microenvironment. First, from a cell‐type‐specific perspective, CD74's function fundamentally differs across various immune cell types. In the B cells we studied, CD74 acts as a chaperone protein for MHC class II molecules, and its core function is to facilitate efficient antigen presentation, a critical step for initiating an adaptive immune response. This function is distinctly different from CD74's role in macrophages or dendritic cells, where it often acts as a receptor for molecules like MIF, activating downstream signaling pathways to mediate immunosuppressive effects.^[^ ^45^ ^]^ Second, the tumor microenvironment is also crucial. In the unique tumor‐associated TLS microenvironment of PSCC, CD74⁺ B cells are activated to support antitumor immunity. Research has shown that the intracellular domain of CD74 plays an important role as a transcriptional regulator in healthy B cells, where it specifically binds to essential genes to finely regulate related pathways. However, in malignant CLL cells, this fine control over key pathways is abolished, leading to abnormal cellular behavior.^[^ ^17^ ^]^

Given that TLSs are dynamic, evolving entities, their cellular composition is expected to continuously change throughout development.^[^ ^46^, ^47^ ^]^ To explore these shifts, we performed a pseudotime analysis of CD74⁺ B cells. Our findings suggested that during differentiation, these cells progressively matured into plasma cells, which was consistent with previous studies demonstrating the ultimate differentiation of mature B cells into plasma cells that produce high‐affinity IgG and IgA antibodies.^[^ ^29^, ^48^, ^49^ ^]^ Notably, the gene expression profiles of CD74⁺ B cells undergo significant alterations during this maturation process: genes related to proliferation, migration, and the immune response decrease in expression, whereas the expression of those associated with apoptosis increases. Interestingly, our analysis revealed that early stage CD74⁺ B cells show significant aggregation within TLSs, despite also being present in nTLS regions. To further investigate the functional disparities between CD74⁺ B cells in these different regions, we conducted a comparative analysis. While the gene expression profiles of CD74⁺ B cells in both TLS and nTLS regions were generally similar, the average expression levels in TLSs were markedly higher, suggesting that the clustering of CD74⁺ B cells within TLSs likely triggered an enhanced immune cascade, thereby potentiating the immune response. These findings provide valuable insight into the role of CD74⁺ B cells in the TLS microenvironment and their contribution to immune activation in PSCC tumors. As B cells are a predominant component of TLSs, their aggregation likely amplifies key immune functions, including antigen presentation, T‐cell activation, and antibody production, thereby eliciting a more potent immune response.^[^ ^27^, ^50^ ^]^ With respect to the relationship between T cells and TLSs, studies have indicated that T cells tend to accumulate early in the TLSs of various cancer tissues. For example, in ovarian cancer, early accumulation of CD4⁺ T cells and CD8⁺ T cells within TLSs enhances the antitumor immune response.^[^ ^51^, ^52^ ^]^ However, our study revealed that in PSCC, the primary interaction of B cells is with naive T cells rather than with CD4⁺ or CD8⁺ T cells. This finding raises an important question: what is the functional role of CD74⁺ B cells in the TLSs of PSCC tissues? Specifically, how do these cells interact with naive T cells to activate immune responses?

Naive T cells, as precursors to effector T cells, are critical in initiating immune responses, especially following antigen presentation.^[^ ^53^, ^54^ ^]^ Previous studies have highlighted their essential role in maintaining immune surveillance and triggering immune activation. Our findings suggest that the accumulation of naive T cells in PSCC tissues may play a distinctive role in orchestrating early immune responses. Within TLSs, CD74⁺ B cells interact with naive T cells via the *HLA‐DRA* ligand, specifically engaging the CD4 receptors of naive T cells. This interaction activates proliferation, differentiation, and immune‐response‐related transcription factors in naive T cells, significantly amplifying the immune response. It is well‐known that naïve T cells, upon activation by B cells, differentiate into mature CD4⁺ or CD8⁺ T cells. We observed that in the PSCC microenvironment, CD74⁺ B cells primarily interact with naïve T cells, providing a direct source for the activation and differentiation of mature CD4⁺/CD8⁺ T cells within TLS. This finding complements existing research on the presence of these mature T cells in TLS and fills a gap in our understanding of the early stages of the TLS immune response. Notably, early stage CD74⁺ B cells within TLSs highly express key antigen‐presenting genes, such as *CD74* and *HLA‐DRA*. *CD74*, in particular, acts as a marker of these B cells and functions as a crucial MHC class II molecular chaperone, facilitating antigen presentation and enhancing T‐cell accumulation and function within the tumor microenvironment.^[^ ^19^, ^55^, ^56^ ^]^ These distinctive features of early stage CD74⁺ B cells suggest that their interactions with naive T cells may be pivotal in shaping the immune response in PSCC, thereby promoting stronger immune activation and contributing to antitumor immunity. The various phenomena and evidence from our study, combined with the finding in Figure 2 that the immune response function of CD74⁺ B cells is more closely aligned with the characteristics of germinal center B cells and memory B cells, form a strong logical loop. It is precisely by leveraging their high‐efficiency antigen presentation and T‐cell activation capabilities, similar to those of germinal center and memory B cells, that CD74⁺ B cells explain their critical role in TLS formation and maintenance. Specifically, the significant enrichment of these cells in PSCC TLSs is a key step in TLS formation itself. Furthermore, through their potential for plasma cell differentiation as revealed by pseudotime analysis, and their critical interaction with naïve T cells within TLSs, they drive a sustained immune response, which is consistent with the function of germinal center B cells. It is through this continuous antigen presentation and T‐cell activation that CD74⁺ B cells are able to maintain the TLS as a mature, functional structure, thereby effectively initiating and continuously driving antitumor immunity within the tumor.

In summary, our findings underscore the pivotal role of CD74⁺ B cells within the TLSs of PSCC tissues, particularly in potentiating the activation and functional maturation of naive T cells. The distinct localization of CD74⁺ B cells in TLSs and their capacity to amplify naive T‐cell functions suggest that these cells are integral to orchestrating early immune responses, positioning them as compelling therapeutic targets for enhancing antitumor immunity in PSCC. While this study provides novel insights into the immune landscape of PSCC tissues, particularly the pivotal role of CD74⁺ B cells within TLSs, several aspects warrant further investigation. For example, although our study revealed a significant correlation between TLS and PSCC prognosis, more comprehensive experimental studies are needed to validate a causal relationship. Furthermore, expanding the sample size and incorporating longitudinal clinical data would help to more accurately assess the clinical relevance of CD74⁺ B cells as a therapeutic target. More in‐depth mechanistic research is required in the future to further explore these dimensions, thereby deepening our understanding of immune regulatory mechanisms within the tumor microenvironment and ultimately validating the therapeutic potential of CD74⁺ B cells in PSCC.

## 4 Experimental Section

### Sample Collection

All the PSCC patient samples used in this study were obtained from the Department of Urology at Sun Yat‐sen University Cancer Center, Guangdong Province. FFPE samples for spatial transcriptomics and fresh tissues for scRNA‐seq were preserved in the appropriate preservation solutions and immediately sent for sequencing. Bulk RNA‐seq samples were stored at −80 °C immediately after preparation. The samples for staining were dehydrated, paraffin‐embedded, and stored at 4 °C. Written informed consent was obtained from all patients or their relatives prior to their participation in the study. All study procedures were conducted in accordance with the ethical standards of the institutional review board and approved by the Ethics Committee of Shenzhen People's Hospital (Approval No. LL‐KY‐2024045‐01). The study was conducted following the principles outlined in the Declaration of Helsinki.

### CosMx Spatial Molecular Imager for RNA (CosMx RNA‐SMI)

In accordance with the method described by Love et al.,^[^ ^57^ ^]^ FFPE tissue sections from 18 PSCC patients samples were mounted onto CITOGLAS slides (catalog no. 158105W) and baked at 60 °C overnight to improve tissue adherence to the slide. Patient clinical characteristics are detailed in Table S1 (Supporting Information). The slides were subjected to heat‐induced epitope retrieval (HIER) using ER1 epitope retrieval buffer (Leica Biosystems) at 100 °C for 15 min. Following HIER, the tissue sections were treated with proteinase K (diluted in ACD Protease Plus, Advanced Cell Diagnostics Inc.) for 30 min. The slides were then washed with DEPC‐treated water and incubated with diluted fiducials (Bangs Laboratory Inc.) in 2× SSCT for 5 min. Excess fiducials were rinsed with 1× phosphate‐buffered saline (PBS), and the tissue sections were fixed with 10% neutral‐buffered formalin for 5 min. After fixation, the sections were washed with Tris‐glycine buffer and PBS and blocked with 100 mm NHS‐acetate (Thermo Fisher Scientific) in NHS‐acetate buffer for 15 min at room temperature. The sections were then washed with 2× SSC and covered with an adhesive secure seal hybridization chamber (Grace Bio‐Labs).

After 2 min of incubation at 95 °C, NanoString ISH probes (including 980 plex and custom probes) were prepared and placed on ice. The probe mixture [1 nm 980 plex ISH probes, 1 nm custom probes, 10 nm attenuation probes, 1× buffer R, and SUPERase·In (0.1 U µL^−1^; Thermo Fisher Scientific) in DEPC H_2_O] was pipetted into the hybridization chamber, and hybridization was carried out at 37 °C overnight. After hybridization, the slides were washed with 2× SSCT and then with 50% formamide and 2× SSC at 37 °C for 25 min, followed by two washes with 2× SSC at room temperature for 2 min each. Blocking was performed with 100 mm NHS‐acetate in the dark for 15 min, and the slides were prepared for loading onto the CosMx SMI instrument.

The CosMx SMI instrument provided the RNA readout. After the air bubbles were removed with Reporter Wash Buffer, the flow cell was prescanned, and areas of interest were selected using 0.51 × 0.51 mm fields of view (FOVs) corresponding to regions of interest identified by H&E staining of adjacent sections. The RNA readout was obtained by flowing 100 µL of Reporter Pool 1 into the flow cell, followed by 15 min of incubation. Unbound probes were removed by washing with 1 mL of Reporter Wash Buffer, and Imaging Buffer was added for imaging. Nine Z‐stack images (0.8 µm step size) were captured from each FOV. After RNA readout, the tissue sections were incubated with a fluorophore‐conjugated antibody cocktail against CD298/B2M (488 nm channel), PanCK (532 nm channel), CD45 (594 nm channel), CD68 (647 nm channel), and DAPI for 2 h. Unbound antibodies and DAPI stain were removed by washing with Reporter Wash Buffer, and Imaging Buffer was added to capture nine Z‐stack images across the five channels (four antibodies and DAPI). The imaging data were processed using the AtoMx Spatial Informatics Platform.

### Cell Segmentation and RNA Transcript Imaging Generation from the CosMx SMI Data

Cell segmentation and RNA transcript imaging were facilitated using machine learning algorithms.^[^ ^58^ ^]^ Initially, the cellular boundaries were delineated on the basis of the Z‐stack images after immunofluorescence and DAPI staining. The transcripts were subsequently spatially assigned to specific cellular locations and subcellular compartments. By integrating the positional data of target transcripts with the boundaries established from the segmentation process, the transcriptomes of individual cells were constructed. Finally, Napari image viewer was employed to overlay the segmented cell contours with the positions of the detected RNA molecules to generate a composite image.^[^ ^59^ ^]^ Each immunofluorescent marker was assigned a distinct color with appropriate contrast adjustments, culminating in the final visual output.

### Identification of the TLS Region

After cell segmentation (Figure S9A, Supporting Information), unsupervised dimensionality reduction, clustering, and cell annotation were performed. Since TLSs contained a high density of B cells, T cells, and dendritic cells, B cell, NK and T cell populations, and DCs in the spatial transcriptomics data of PSCC tissue (Figure S9B, Supporting Information) were identified. Based on this identification, TLS regions were defined using a density‐based clustering algorithm with the DBSCAN function from scikit‐learn (v1.5.2).^[^ ^59^ ^]^ The clustering was based on the expression of cell type‐specific biomarker genes for B cells, NK cells, T cells, and DCs, with parameters set as eps = 0.08 and min_samples = 100. Clusters containing more than 100 cells were defined as TLS regions (Figure S9C, Supporting Information). After comparison of the algorithm‐derived regions with H&E images of control samples by a pathology expert, the regions were confirmed to be TLS‐like regions (Figure S9D, Supporting Information). Following this, the number of cells was calculated within each TLS for each sample and the samples were categorized into high‐ and low‐TLS score groups based on the median cell count. TLS score grouping was based on the cohort‐specific median of the cumulative TLS area per patient, as measured from H&E‐stained PSCC tissue sections scanned by the HALO platform (i.e., the sum of all TLS regions within a single section). Patients were categorized into high‐ and low‐TLS score groups according to whether their cumulative TLS area was above or below this median, which was 5.16 mm^2^ in the internal cohort (*n* = 152) and 4.76 mm^2^ in the external cohort (*n* = 63). Patient clinical information and comparisons are detailed in Tables S2–S4 (Supporting Information).

### ScRNA‐Seq

Twenty‐one fresh PSCC samples were placed in enzyme solution and incubated at 37 °C to promote tissue dissociation. Patient clinical characteristics are detailed in Table S5 (Supporting Information). After dissociation, the cells were processed by pipetting and filtration to obtain uniform single‐cell suspensions, followed by centrifugation and washing to remove excess enzymes and debris. The cell number and viability were assessed using a cell counter, ensuring a viability rate of greater than 90%. After the concentration of the cell suspension was adjusted, microfluidic technology on the 10× Genomics Chromium platform was used to mix the cells with barcode‐labeled beads to form water‐in‐oil droplets (GEMs). During this process, the cells were lysed, releasing mRNA, which then bound to the barcodes on the beads, forming single‐cell GEMs. Once the droplets ruptured, the released mRNA underwent reverse transcription to generate cDNA, followed by library construction. Finally, the libraries were sequenced using the Illumina platform to obtain high‐throughput single‐cell transcriptomic data.

### ScRNA‐Seq Data Processing

The scRNA‐seq data were processed by aligning the raw reads to the human genome (GRCh38) using CellRanger and generated a feature barcode matrix. Initial sequencing generated 200 221 cells. After quality control, which excluded cells with fewer than 500 genes, more than 5000 genes, or mitochondrial gene content exceeding 20%, 150 540 high‐quality cells were retained for downstream analysis. Data normalization was then performed using the Seurat package, with the NormalizeData and ScaleData functions applied to minimize technical noise. Batch effects were corrected using the Harmony function. Principal component analysis was conducted for dimensionality reduction, followed by unsupervised clustering using the FindNeighbors and FindClusters functions. The clustering results were visualized using UMAP or t‐SNE. Finally, differential gene expression analysis was conducted using the FindAllMarkers function, and cell populations were annotated on the basis of classical marker genes and gene combinations reported in the literature.

### Bulk RNA‐Seq

Total RNA was extracted from the tissues of 55 PSCC patients using TRIzol reagent according to standard procedures. Patient clinical characteristics and a comparison of clinical characteristics across the three sequencing methods are presented in Tables S6 and S7 (Supporting Information). The extracted RNA was assessed using an Agilent 2100 bioanalyzer to ensure its integrity (RIN value ≥7). Samples that met the quality criteria were used for mRNA enrichment from 1 µg of total RNA with the NEB Next Poly(A) mRNA Magnetic Isolation Module (E7490L, NEB), followed by library preparation using the KAPA RNA Library Preparation Kit (Illumina). The constructed cDNA libraries were sequenced on an Illumina NovaSeq 6000 platform with 150 bp paired‐end reads using the NovaSeq S4 reagent kit.

### Bulk RNA‐Seq Data Processing

The raw sequencing data were first subjected to quality control and adapter trimming using fastp. The processed reads were then aligned to the hg38 reference genome using Bowtie 2. After alignment, RNA‐seq by expectation‐maximization was employed to generate both raw counts and transcripts per million values, which were subsequently used for downstream analysis.

### Kaplan–Meier Survival Analysis

The samples were grouped on the basis of the TLS score, specific cell signature score, or expression levels of certain genes. The survival curves were plotted using the R package survival (version 3.5), and the survival probabilities for each group were calculated. Log‐rank tests were then applied to assess significant differences in survival between groups.

### GO Enrichment Analysis

In the R package Seurat (version 5.1.0), the FindAllMarkers function was used to identify differentially expressed genes (DEGs) via the Wilcoxon rank‐sum test with Bonferroni's correction. The selection criteria were a *p* value of less than 0.05 and a log_2_ fold change (log_2_FC) greater than 0.2. Next, GO enrichment analysis of the DEGs was performed using the enrichGO function in the clusterProfiler package (version 3.18.1).

### AUCell

AUCell (version 1.26.0) was used to perform enrichment analysis of the data by calculating the activity score for each cell. DEGs were then identified using the Wilcoxon rank‐sum test with the Seurat package, with the Bonferroni correction applied to control the false discovery rate (FDR) to be less than 0.01 and a log_2_FC greater than 0.8.

### CIBERSORT

CIBERSORT was used to estimate the signature scores of B‐cell subtypes in PSCC patients, utilizing gene expression profiles from the bulk RNA data. The samples were classified into two groups on the basis of the median value of the B‐cell subtype signature score. The log‐rank test was performed for survival analysis, and Kaplan–Meier curves were generated for each group. A value of *p* < 0.05 was considered to indicate statistical significance.

### ssGSEA

Cell‐type‐specific gene signatures were generated with Seurat v5.1.0 by applying FindAllMarkers (two‐sided Wilcoxon test) to each annotated cluster; all genes meeting the criteria of Benjamini–Hochberg‐adjusted *p* < 0.05 and absolute log_2_ fold change > 0.1 were retained, and these marker sets were used as input for the GSVA R package to calculate single‐sample GSEA enrichment scores.

### Spatial Transcriptomics Pseudotime Analysis

In the Python (3.8.13) environment, data analysis was conducted using Palantir (1.3.3) and CellRank2 (2.0.4) for spatial transcriptomics. First, Palantir was utilized to infer cellular pseudotime and reconstruct the differentiation trajectories, followed by an analysis of gene expression dynamics along these trajectories. The CellRank2 algorithm was subsequently applied to identify key driver genes that were significantly associated with each cell lineage, leveraging the predominant pseudotime. The results were visualized using Matplotlib (3.7.2) and Seaborn (0.13.2).

### Single‐Cell Mapping onto Spatial Distribution

Tangram (version 1.0.4) was used to map the single‐cell and spatial transcriptomics data for scRNA‐seq. The pp_adatas function was subsequently used for data preprocessing, and deconvolution analysis was performed using the map_cells_to_space function. The deconvolution results were used to annotate the cell types from the scRNA‐seq data, identifying the spatial distribution of single cells.

### Transcription Factor (TF) Regulatory Network Inference

The TF regulatory network was inferred at the single‐cell level using pySCENIC (version 0.12.1). On the basis of the hg38 reference genome and TF binding site information from the Cistrome database, candidate regulatory sequences were defined as the regions 500 bp upstream to 100 bp downstream of the transcription start site. The GRNBoost2 algorithm was used to calculate the coexpression weight between the genes and TFs, selecting significant regulatory relationships (adj. *p* < 0.01) to construct cell‐type‐specific regulatory networks (regulons). The AUCell algorithm was then applied to quantify regulon activity at the single‐cell level. The spatial distribution of TF activity was mapped to the tissue in situ coordinates using SpaceRanger (v2.0). The spatial heterogeneity of the regulatory modules was visualized via heatmaps and network topology diagrams.

### MISTy

The mistyR package in R (version 1.10.0) was applied for neighborhood analysis. First, the data were preprocessed with the create_initial_view function to retain informative features, ensuring the validity of the subsequent analyses. Then, the visualization and analysis of spatial transcriptomic data were enhanced using add_paraview, which incorporated spatial location and smoothing techniques. Finally, run_misty was employed to investigate the spatial relationships between cells, revealing their clustering and spatial distribution characteristics.

### Cell–Cell Communication Analysis

Using the CellChat package in R (version 2.1.0), cell–cell communication networks were inferred on the basis of the ligand–receptor pair database. The computeCommunProb and filterCommunication functions were used to predict communication networks, after which computeCommunProbPathway was employed to calculate communication probabilities at the signaling pathway level. Finally, the aggregateNet function was employed to generate the aggregated cell communication network.

### H&E Staining, Immunohistochemistry (IHC), and mIF

This study utilized FFPE sections for H&E staining, IHC, and mIF experiments. The samples were fixed in 10% neutral‐buffered formalin, dehydrated through graded ethanol, deparaffinized with xylene, embedded in paraffin, and then cut into 5 µm thick sections. For H&E staining, the sections were deparaffinized and rehydrated, and then treated with hematoxylin (nuclei) and eosin (cytoplasmic differentiation), followed by dehydration, clearing with xylene, and mounting. IHC was performed via heat‐induced antigen retrieval, blocking endogenous peroxidase activity with 3% H_2_O_2_, and blocking nonspecific binding with 5% bovine serum albumin. The sections were then incubated with primary antibodies followed by HRP‐conjugated secondary antibodies. After DAB color development, the sections were counterstained with hematoxylin and mounted. The staining results were analyzed using the HALO platform (v4.0). The IRISKit HyperView mTSA Kit was used for mIF. After primary and secondary antibody labeling, TSA–HRP and fluorescent dyes were applied for signal amplification. After each round of staining, the antibody complexes were removed with stripping buffer, and three targets were cyclically labeled. Finally, DAPI was used to counterstain the nuclei, and the sections were mounted before imaging with a fluorescence microscope. The catalog numbers and dilutions of the antibodies used are listed in Table S8 (Supporting Information).

### Immunotherapy Cohort Sample Collection

In accordance with previous studies,^[^ ^60^ ^]^ patients were recruited from Sun Yat‐sen University Cancer Center and received neoadjuvant treatment with the TNT regimen (ClinicalTrials.gov identifier: NCT04475016). Treatment cycles were repeated every three weeks, up to a maximum of four cycles, or until dose‐limiting toxicity, disease progression, or patient withdrawal occurred. All the samples were collected before treatment. The primary endpoint was the pCR rate. pCR was defined as the absence of residual invasive tumor cells in resected tumor sites (ypTis/T0N0M0). The secondary endpoints included the objective response rate, defined as the proportion of patients who achieved a best response of CR or PR; PFS, defined as the time from the initiation of neoadjuvant TNT to disease progression (including local and/or distant progression) or death from any cause; OS, defined as the time from the initiation of TNT to death from any cause; and safety. Clinical response was evaluated every two cycles by enhanced CT imaging or MRI and independently reviewed by two blinded radiologists on the basis of the RECIST version 1.1 criteria.

### CD74 B‐Cell Sorting and Cultivation

Fresh tumor tissues from PSCC patients were collected and mechanically dissociated into a single‐cell suspension. The cells were then filtered through a 70 µm cell strainer to remove debris and ensure uniformity. Next, the cells were stained with a panel of antibodies targeting surface markers, including CD74, CD20, and CD45, to distinguish different cell populations, particularly CD74^+^ and CD74^−^ B cells. The antibodies used were CD20 (Proteintech, PE‐FCA65575), CD45 (Proteintech, FITC‐98117), and CD74 (Biolegend, 326812). The flow cytometry gating strategy involved identifying viable lymphocytes based on forward scatter (FSC‐H) and side scatter (SSC‐A), followed by CD45 staining to exclude nonimmune cells. CD74^+^ and CD74^−^ populations were further separated based on CD20 expression. The sorted cell populations were then collected and cultured in vitro for further analysis.

The two sorted cell populations were seeded at a density of 1.5 × 10⁶ cells mL^−1^ in complete RPMI 1640 medium containing 10% fetal bovine serum. They were then stimulated with a cocktail of reagents to induce B‐cell maturation and plasma cell differentiation, which included: IL‐21 (50 ng mL^−1^; BioLegend), BAFF (10 ng mL^−1^; BioLegend), IL‐10 (250 ng mL^−1^; BioLegend), and R848 (1 µg mL^−1^; Invivogen). Cultures were maintained in a 37 °C, 5% CO_2_ incubator, with half of the fresh medium being replaced every 2–3 days to sustain cell viability.^[^ ^61^ ^]^ To examine the differentiation of B cells into plasma cells, cells were collected on days 0, 3, 7, and 10. The day 0 samples were collected immediately after sorting, while cells from subsequent time points were harvested from the cultures, washed with PBS, and processed to obtain cell pellets or lysates for analysis.

### Western Blot

Cell pellets from CD74^+^ and CD74^−^ B cells collected at different time points (days 0, 3, 7, and 10) were lysed in RIPA buffer containing protease and phosphatase inhibitors. After protein concentration was quantified using a BCA assay, equal amounts of protein were separated by electrophoresis on a 10% sodium dodecyl sulfate–polyacrylamide gel electrophoresis gel and then transferred to a PVDF membrane. The membrane was blocked with 5% nonfat milk for 1 h at room temperature. Subsequently, it was incubated overnight at 4 °C with the following primary antibodies: anti‐CD38 (1:2000, abcam, ab108403), anti‐CD138 recombinant rabbit monoclonal antibody (1:2000, Thermo Fisher Scientific, MA5‐32600), anti‐PRDM1/Blimp‐1 (1:1000, CST, 9115S), and anti‐XBP‐1s (1:1000, CST, 40435S). Anti‐GAPDH (1:10 000, Proteintech, HRP‐60004) was used as a loading control. After washing with PBST, the corresponding secondary antibodies, anti‐rabbit (1:1000, CST, #7074S) and anti‐mouse (1:1500, CST, #7076), were incubated for 1 h at room temperature. Finally, protein bands were visualized using an ECL detection kit.

### Statistical Analysis

For two‐group comparisons of continuous variables (e.g., signature scores, AUCell/ssGSEA scores, cell proportions), Student's *t*‐test was used when both groups satisfied normality and variance homogeneity assumptions; otherwise, the Wilcoxon rank‐sum test was applied. The same criteria were applied in MISTy analyses comparing predictor importances between groups. Survival analyses were performed using Kaplan–Meier estimates with the log‐rank test, with groups defined by the median of the corresponding score. Unless otherwise specified, all tests were two‐sided, statistical significance was set at *p* < 0.05, and *p* values were adjusted for multiple comparisons using the Benjamini–Hochberg FDR method where applicable. *p* > 0.05 indicated no significant difference, * indicated *p* < 0.05, ** indicated *p* < 0.01, and *** indicated *p* < 0.001.

## Conflict of Interest

The authors declare no conflict of interest.

## Author Contributions

T.X., C.D., and J.L. contributed equally to this work. T.X., C.D., and J.L. contributed equally to the conceptualization and methodology of the study. R.Y., J.L., X.L., and D.W. were responsible for data curation. X.H., X.X., J.J., and D.C. contributed to the formal analysis and software development. J.L., H.T., and Y.L. provided critical resources. The original draft of the paper was prepared by T.X., C.D., and Z.Z. The paper was reviewed and edited by B.C., H.H., and Z.L. Funding acquisition was provided by B.C., H.H., and Z.L. All the authors read and approved the final version of the paper.

## Supporting information



Supporting Information

Supplemental Tables 1‐8

## Data Availability

The data that support the findings of this study are available from the corresponding author upon reasonable request.

## References

[1] A. Thomas , A. Necchi , A. Muneer , M. Tobias‐Machado , A. T. H. Tran , A. S. Van Rompuy , P. E. Spiess , M. Albersen , Nat. Rev. Dis. Primers 2021, 7, 11. 33574340 10.1038/s41572-021-00246-5

[2] D. R. Dickstein , C. R. Edwards , E. J. Lehrer , E. S. Tarras , M. Gallitto , J. Sfakianos , M. D. Galsky , R. Stock , J. D. Safer , B. R. S. Rosser , D. C. Marshall , Nat. Rev. Urol. 2023, 20, 332. 37217695 10.1038/s41585-023-00778-3 PMC10389287

[3] L. Elst , G. Philips , K. Vandermaesen , A. Bassez , F. Lodi , M. T. A. Vreeburg , O. R. Brouwer , R. Schepers , T. Van Brussel , S. K. Mohanty , A. V. Parwani , L. Spans , I. Vanden Bempt , G. Jacomen , M. Baldewijns , D. Lambrechts , M. Albersen , Eur. Urol. 2024, 86, 114. 38670879 10.1016/j.eururo.2024.03.038

[4] A. Necchi , P. E. Spiess , T. Costa de Padua , R. Li , P. Grivas , R. S. P. Huang , D. I. Lin , N. Danziger , J. S. Ross , J. M. Jacob , R. A. Sager , A. Basnet , G. Li , R. P. Graf , D. C. Pavlick , G. Bratslavsky , JAMA Network Open 2023, 6, 2348002. 10.1001/jamanetworkopen.2023.48002 PMC10753400 38150257

[5] H. Moch , M. B. Amin , D. M. Berney , E. M. Compérat , A. J. Gill , A. Hartmann , S. Menon , M. R. Raspollini , M. A. Rubin , J. R. Srigley , P. Hoon Tan , S. K. Tickoo , T. Tsuzuki , S. Turajlic , I. Cree , G. J. Netto , Eur. Urol. 2022, 82, 458. 35853783 10.1016/j.eururo.2022.06.016

[6] L. Xiong , X. Shan , H. Ma , S. Guo , J. Liu , X. Chen , W. Meng , B. Guo , L. Jiang , R. Yan , X. An , Y. Shi , Y. Zhang , T. Xue , L. Wei , D. Xu , Z. Zhang , Z. Qin , K. Yao , Y. Li , P. E. Spiess , L. Hu , N. Xing , H. Han , J. Natl. Compr. Cancer Network 2024, 23, 247074. 10.6004/jnccn.2024.7074 39705804

[7] E. Gambale , S. Fancelli , E. Caliman , M. C. Petrella , L. Doni , S. Pillozzi , L. Antonuzzo , J. Immunother. Cancer 2022, 10, 003540. 10.1136/jitc-2021-003540 PMC8804682 35101944

[8] T. Huang , X. Cheng , J. Chahoud , A. Sarhan , P. Tamboli , P. Rao , M. Guo , G. Manyam , L. Zhang , Y. Xiang , L. Han , X. Shang , P. Deng , Y. Luo , X. Lu , S. Feng , M. M. Ferrer , Y. Alan Wang , R. A. DePinho , C. A. Pettaway , X. Lu , Nat. Commun. 2020, 11, 2124. 32358507 10.1038/s41467-020-15980-9 PMC7195486

[9] R. Cabrita , M. Lauss , A. Sanna , M. Donia , M. Skaarup Larsen , S. Mitra , I. Johansson , B. Phung , K. Harbst , J. Vallon‐Christersson , A. van Schoiack , K. Lövgren , S. Warren , K. Jirström , H. Olsson , K. Pietras , C. Ingvar , K. Isaksson , D. Schadendorf , H. Schmidt , L. Bastholt , A. Carneiro , J. A. Wargo , I. M. Svane , G. Jönsson , Nature 2020, 577, 561. 31942071 10.1038/s41586-019-1914-8

[10] J. L. Teillaud , A. Houel , M. Panouillot , C. Riffard , M. C. Dieu‐Nosjean , Nat. Rev. Cancer 2024, 24, 629. 39117919 10.1038/s41568-024-00728-0

[11] S. T. Paijens , A. Vledder , M. de Bruyn , H. W. Nijman , Cell Mol. Immunol. 2021, 18, 842. 33139907 10.1038/s41423-020-00565-9 PMC8115290

[12] D. M. Xu , X. Y. Zhuang , H. L. Ma , Z. S. Li , L. C. Wei , J. H. Luo , H. Han , Cancer Med. 2024, 13, 70025. 10.1002/cam4.70025 PMC11246611 39003681

[13] W. H. Fridman , M. Meylan , G. Pupier , A. Calvez , I. Hernandez , C. Sautès‐Fridman , Immunity 2023, 56, 2254. 37699391 10.1016/j.immuni.2023.08.009

[14] L. Esparcia‐Pinedo , N. Romero‐Laorden , A. Alfranca , Front. Immunol. 2023, 14, 1231315. 37622111 10.3389/fimmu.2023.1231315 PMC10445545

[15] W. H. Fridman , M. Meylan , F. Petitprez , C. M. Sun , A. Italiano , C. Sautès‐Fridman , Nat. Rev. Clin. Oncol. 2022, 19, 441. 35365796 10.1038/s41571-022-00619-z

[16] D. Starlets , Y. Gore , I. Binsky , M. Haran , N. Harpaz , L. Shvidel , S. Becker‐Herman , A. Berrebi , I. Shachar , Blood 2006, 107, 4807. 16484589 10.1182/blood-2005-11-4334

[17] K. David , G. Friedlander , B. Pellegrino , L. Radomir , H. Lewinsky , L. Leng , R. Bucala , S. Becker‐Herman , I. Shachar , Cell Rep. 2022, 41, 111572. 36323260 10.1016/j.celrep.2022.111572

[18] H. Bergmann , M. Yabas , A. Short , L. Miosge , N. Barthel , C. E. Teh , C. M. Roots , K. R. Bull , Y. Jeelall , K. Horikawa , B. Whittle , B. Balakishnan , G. Sjollema , E. M. Bertram , F. Mackay , A. J. Rimmer , R. J. Cornall , M. A. Field , T. D. Andrews , C. C. Goodnow , A. Enders , J. Exp. Med. 2013, 210, 31. 23267016 10.1084/jem.20121076 PMC3549710

[19] E. Bonnin , M. Rodrigo Riestra , F. Marziali , R. Mena Osuna , J. Denizeau , M. Maurin , J. J. Saez , M. Jouve , P. E. Bonté , W. Richer , F. Nevo , S. Lemoine , N. Girard , M. Lefevre , E. Borcoman , A. Vincent‐Salomon , S. Baulande , H. D. Moreau , C. Sedlik , C. Hivroz , A. M. Lennon‐Duménil , J. Tosello Boari , E. Piaggio , Nat. Commun. 2024, 15, 3749. 38702311 10.1038/s41467-024-47981-3 PMC11068745

[20] A. Nakamura , F. Zeng , S. Nakamura , K. T. Reid , E. Gracey , M. Lim , L. Leng , S. Jo , Y. S. Park , M. Kusuda , R. Machhar , S. F. Boroojeni , B. Wu , E. Rossomacha , T. H. Kim , F. Ciccia , J. S. Rockel , M. Kapoor , R. D. Inman , I. Jurisica , S. Q. Crome , R. Bucala , N. Haroon , Sci. Transl. Med. 2021, 13, abg1210. 10.1126/scitranslmed.abg1210 34669443

[21] N. M. Chapman , M. R. Boothby , H. Chi , Nat. Rev. Immunol. 2020, 20, 55. 31406325 10.1038/s41577-019-0203-y

[22] X. Wang , X. Li , J. Zhao , Y. Li , S. R. Shin , G. Ligresti , A. H. M. Ng , J. S. Bromberg , G. Church , D. R. Lemos , R. Abdi , Adv. Mater. 2024, 36, 2308760. 10.1002/adma.202308760 PMC11009051 38306610

[23] S. Hong , Z. Zhang , H. Liu , M. Tian , X. Zhu , Z. Zhang , W. Wang , X. Zhou , F. Zhang , Q. Ge , B. Zhu , H. Tang , Z. Hua , B. Hou , Immunity 2018, 49, 695. 30291027 10.1016/j.immuni.2018.08.012

[24] C. Ma , C. Yang , A. Peng , T. Sun , X. Ji , J. Mi , L. Wei , S. Shen , Q. Feng , Mol. Cancer 2023, 22, 170. 37833788 10.1186/s12943-023-01876-x PMC10571470

[25] Y. Yang , X. Chen , J. Pan , H. Ning , Y. Zhang , Y. Bo , X. Ren , J. Li , S. Qin , D. Wang , M. M. Chen , Z. Zhang , Cell 2024, 187, 4790. 39047727 10.1016/j.cell.2024.06.038

[26] J. C. Wortman , T. F. He , S. Solomon , R. Z. Zhang , A. Rosario , R. Wang , T. Y. Tu , D. Schmolze , Y. Yuan , S. E. Yost , X. Li , H. Levine , G. Atwal , P. P. Lee , C. C. Yu , npj Breast Cancer 2021, 7, 84. 34210991 10.1038/s41523-021-00291-z PMC8249408

[27] T. N. Schumacher , D. S. Thommen , Science 2022, 375, abf9419. 10.1126/science.abf9419 34990248

[28] D. Hao , G. Han , A. Sinjab , L. I. Gomez‐Bolanos , R. Lazcano , A. Serrano , S. D. Hernandez , E. Dai , X. Cao , J. Hu , M. Dang , R. Wang , Y. Chu , X. Song , J. Zhang , E. R. Parra , J. A. Wargo , S. G. Swisher , T. Cascone , B. Sepesi , A. P. Futreal , M. Li , S. M. Dubinett , J. Fujimoto , L. M. Solis Soto , I. I. Wistuba , C. S. Stevenson , A. Spira , S. Shalapour , H. Kadara , et al., Cancer Discovery 2022, 12, 2626. 36098652 10.1158/2159-8290.CD-21-1658 PMC9633381

[29] E. Alaterre , S. Ovejero , C. Bret , L. Dutrieux , D. Sika , R. F. Perez , M. Espéli , T. Fest , M. Cogné , J. I. Martin‐Subero , P. Milpied , G. Cavalli , J. Moreaux , Blood 2024, 144, 496. 38643512 10.1182/blood.2023023237 PMC11406183

[30] S. Trowitzsch , R. Tampé , Annu. Rev. Biophys. 2020, 49, 135. 32004089 10.1146/annurev-biophys-121219-081643

[31] A. J. Voss , E. Korb , Trends Genet. 2025, 41, P506. 10.1016/j.tig.2025.01.003 PMC12167159 39984351

[32] L. Zheng , L. Ren , A. Kouhi , L. A. Khawli , P. Hu , H. R. Kaslow , A. L. Epstein , Clin. Cancer Res. 2020, 26, 3694. 32273277 10.1158/1078-0432.CCR-19-3417

[33] M. M. Lee‐Sundlov , R. T. Burns , T. O. Kim , R. Grozovsky , S. Giannini , L. Rivadeneyra , Y. Zheng , S. H. Glabere , W. H. A. Kahr , R. Abdi , J. M. Despotovic , D. Wang , K. M. Hoffmeister , Blood 2021, 138, 2408. 34324649 10.1182/blood.2020008238 PMC8662070

[34] Y. Y. Feng , M. Tang , M. Suzuki , C. Gunasekara , Y. Anbe , Y. Hiraoka , J. Liu , H. Grasberger , M. Ohkita , Y. Matsumura , J. Y. Wang , T. Tsubata , J. Immunol. 2019, 202, 2546. 30867238 10.4049/jimmunol.1800443

[35] C.‐C. Chen , B.‐R. Chen , Y. Wang , P. Curman , H. A. Beilinson , R. M. Brecht , C. C. Liu , R. J. Farrell , J. de Juan‐Sanz , L.‐M. Charbonnier , J. Exp. Med. 2021, 218, 20201708. 10.1084/jem.20201708 PMC8155808 34033676

[36] J.‐X. Lin , M. Ge , C.‐y. Liu , R. Holewinski , T. Andresson , Z.‐X. Yu , T. Gebregiorgis , R. Spolski , P. Li , W. J. Leonard , Nat. Commun. 2024, 15, 7372. 39191751 10.1038/s41467-024-50925-6 PMC11349758

[37] J. E. Ramis‐Zaldivar , B. Gonzalez‐Farré , O. Balagué , V. Celis , F. Nadeu , J. Salmerón‐Villalobos , M. Andrés , I. Martin‐Guerrero , M. Garrido‐Pontnou , A. Gaafar , M. Suñol , C. Bárcena , F. Garcia‐Bragado , M. Andión , D. Azorín , I. Astigarraga , M. Sagaseta de Ilurdoz , C. Sábado , S. Gallego , J. Verdú‐Amorós , R. Fernandez‐Delgado , V. Perez , G. Tapia , A. Mozos , M. Torrent , P. Solano‐Páez , A. Rivas‐Delgado , I. Dlouhy , G. Clot , A. Enjuanes , et al., Blood 2020, 135, 274. 31738823 10.1182/blood.2019002699 PMC6978155

[38] B. J. Laidlaw , J. G. Cyster , Nat. Rev. Immunol. 2021, 21, 209. 33024284 10.1038/s41577-020-00446-2 PMC7538181

[39] C. T. Berry , X. Liu , A. Myles , S. Nandi , Y. H. Chen , U. Hershberg , I. E. Brodsky , M. P. Cancro , C. J. Lengner , M. J. May , Cell Rep. 2020, 31, 107474. 32294437 10.1016/j.celrep.2020.03.038 PMC7301411

[40] T. Redmer , M. Raigel , C. Sternberg , R. Ziegler , C. Probst , D. Lindner , A. Aufinger , T. Limberger , K. Trachtova , P. Kodajova , S. Högler , M. Schlederer , S. Stoiber , M. Oberhuber , M. Bolis , H. A. Neubauer , S. Miranda , M. Tomberger , N. S. Harbusch , I. Garces de Los Fayos Alonso , F. Sternberg , R. Moriggl , J. P. Theurillat , B. Tichy , V. Bystry , J. L. Persson , S. Mathas , F. Aberger , B. Strobl , S. Pospisilova , et al., Mol. Cancer 2024, 23, 114. 38811984 10.1186/s12943-024-02022-x PMC11134959

[41] R. K. Perez , M. G. Gordon , M. Subramaniam , M. C. Kim , G. C. Hartoularos , S. Targ , Y. Sun , A. Ogorodnikov , R. Bueno , A. Lu , M. Thompson , N. Rappoport , A. Dahl , C. M. Lanata , M. Matloubian , L. Maliskova , S. S. Kwek , T. Li , M. Slyper , J. Waldman , D. Dionne , O. Rozenblatt‐Rosen , L. Fong , M. Dall'Era , B. Balliu , A. Regev , J. Yazdany , L. A. Criswell , N. Zaitlen , C. J. Ye , Science 2022, 376, abf1970. 10.1126/science.abf1970 PMC9297655 35389781

[42] Z. Zhang , L. Chen , H. Chen , J. Zhao , K. Li , J. Sun , M. Zhou , EBioMedicine 2022, 83, 104207. 35961204 10.1016/j.ebiom.2022.104207 PMC9382263

[43] Z. Wang , B. Wang , Y. Feng , J. Ye , Z. Mao , T. Zhang , M. Xu , W. Zhang , X. Jiao , Q. Zhang , Y. Zhang , B. Cui , J. Immunother. Cancer 2024, 12, 009024. 10.1136/jitc-2024-009024 PMC11308911 39107132

[44] B. Pellegrino , K. David , S. Rabani , B. Lampert , T. Tran , E. Doherty , M. Piecychna , R. Meza‐Romero , L. Leng , D. Hershkovitz , A. A. Vandenbark , R. Bucala , S. Becker‐Herman , I. Shachar , PLoS Biol. 2024, 22, 3002905. 10.1371/journal.pbio.3002905 PMC11623796 39576827

[45] G. Q. Zhu , Z. Tang , R. Huang , W. F. Qu , Y. Fang , R. Yang , C. Y. Tao , J. Gao , X. L. Wu , H. X. Sun , Y. F. Zhou , S. S. Song , Z. B. Ding , Z. Dai , J. Zhou , D. Ye , D. J. Wu , W. R. Liu , J. Fan , Y. H. Shi , Cell Discovery 2023, 9, 25. 36878933 10.1038/s41421-023-00529-z PMC9988869

[46] Y. Zhang , M. Xu , Y. Ren , Y. Ba , S. Liu , A. Zuo , H. Xu , S. Weng , X. Han , Z. Liu , Mol. Cancer 2024, 23, 75. 38582847 10.1186/s12943-024-01980-6 PMC10998345

[47] L. Zhao , S. Jin , S. Wang , Z. Zhang , X. Wang , Z. Chen , X. Wang , S. Huang , D. Zhang , H. Wu , Signal Transduction Targeted Ther. 2024, 9, 225. 10.1038/s41392-024-01947-5 PMC11358547 39198425

[48] C. L. Freeman , J. Noble , M. Menges , R. Villanueva , J. Y. Nakashima , N. B. Figura , R. P. Tonseth , D. W. Idiaquez , L. Skelson , E. C. Smith , J. Abraham‐Miranda , S. Corallo , G. De Avila , O. Castaneda Puglianini , H. D. Liu , M. Alsina , T. Nishihori , K. H. Shain , R. C. Baz , B. J. Blue , A. Grajales‐Cruz , J. M. Koomen , R. M. Atkins , D. K. Hansen , A. Siqueira Silva , J. Kim , Y. Balagurunathan , F. L. Locke , Blood 2024, 145, 1645. 10.1182/blood.2024024965 39652773

[49] G. K. Manakkat Vijay , M. Zhou , K. Thakkar , A. Rothrauff , A. S. Chawla , D. Chen , L. C. Lau , P. H. Gerges , K. Chetal , P. Chhibbar , J. Fan , J. Das , A. Joglekar , L. Borghesi , N. Salomonis , H. Xu , H. Singh , Nat. Immunol. 2024, 25, 1097. 38698087 10.1038/s41590-024-01831-y

[50] C. Hu , W. You , D. Kong , Y. Huang , J. Lu , M. Zhao , Y. Jin , R. Peng , D. Hua , D. M. Kuang , Y. Chen , Gastroenterology 2023, 166, 1069. 38445519 10.1053/j.gastro.2023.10.022

[51] R. A. Chaurio , C. M. Anadon , T. L. Costich , K. K. Payne , S. Biswas , C. M. Harro , C. Moran , A. C. Ortiz , C. Cortina , K. E. Rigolizzo , K. B. Sprenger , J. A. Mine , P. Innamarato , G. Mandal , J. J. Powers , A. Martin , Z. Wang , S. Mehta , B. A. Perez , R. Li , J. Robinson , J. L. Kroeger , T. J. Curiel , X. Yu , P. C. Rodriguez , J. R. Conejo‐Garcia , Immunity 2022, 55, 115. 35021053 10.1016/j.immuni.2021.12.007 PMC8852221

[52] M. Ukita , J. Hamanishi , H. Yoshitomi , K. Yamanoi , S. Takamatsu , A. Ueda , H. Suzuki , Y. Hosoe , Y. Furutake , M. Taki , K. Abiko , K. Yamaguchi , H. Nakai , T. Baba , N. Matsumura , A. Yoshizawa , H. Ueno , M. Mandai , JCI Insight 2022, 7, 157215. 10.1172/jci.insight.157215 PMC9309049 35552285

[53] Y. Chen , Z. Xu , H. Sun , X. Ouyang , Y. Han , H. Yu , N. Wu , Y. Xie , B. Su , Cell Mol. Immunol. 2023, 20, 1023. 37582972 10.1038/s41423-023-01064-3 PMC10468538

[54] B. D. Hale , Y. Severin , F. Graebnitz , D. Stark , D. Guignard , J. Mena , Y. Festl , S. Lee , J. Hanimann , N. S. Zangger , M. Meier , D. Goslings , O. Lamprecht , B. M. Frey , A. Oxenius , B. Snijder , Science 2024, 384, adh8697. 10.1126/science.adh8967 38843327

[55] L. Liu , J. Wang , Y. Wang , L. Chen , L. Peng , Y. Bin , P. Ding , R. Zhang , F. Tong , X. Dong , J. Exp. Clin. Cancer Res. 2024, 43, 128. 38685050 10.1186/s13046-024-03024-9 PMC11059744

[56] Z. Qiu , J. Khalife , P. Ethiraj , C. Jaafar , A. P. Lin , K. N. Holder , J. P. Ritter , L. Chiou , G. Huelgas‐Morales , S. Aslam , Z. Zhang , Z. Liu , S. Arya , Y. K. Gupta , P. L. M. Dahia , R. C. T. Aguiar , Sci. Adv. 2024, 10, adk2091. 10.1126/sciadv.adk2091 PMC11244530 38996030

[57] N. R. Love , C. Williams , E. E. Killingbeck , A. Merleev , M. Saffari Doost , L. Yu , J. D. McPherson , H. Mori , A. D. Borowsky , E. Maverakis , M. Kiuru , Sci. Adv. 2024, 10, adm8206. 10.1126/sciadv.adm8206 PMC11244543 38996022

[58] S. He , R. Bhatt , C. Brown , E. A. Brown , D. L. Buhr , K. Chantranuvatana , P. Danaher , D. Dunaway , R. G. Garrison , G. Geiss , M. T. Gregory , M. L. Hoang , R. Khafizov , E. E. Killingbeck , D. Kim , T. K. Kim , Y. Kim , A. Klock , M. Korukonda , A. Kutchma , Z. R. Lewis , Y. Liang , J. S. Nelson , G. T. Ong , E. P. Perillo , J. C. Phan , T. Phan‐Everson , E. Piazza , T. Rane , Z. Reitz , et al., Nat. Biotechnol. 2022, 40, 1794. 36203011 10.1038/s41587-022-01483-z

[59] R. Dries , Q. Zhu , R. Dong , C. L. Eng , H. Li , K. Liu , Y. Fu , T. Zhao , A. Sarkar , F. Bao , R. E. George , N. Pierson , L. Cai , G. C. Yuan , Genome Biol. 2021, 22, 78. 33685491 10.1186/s13059-021-02286-2 PMC7938609

[60] X. An , S. J. Guo , R. Yan , T. Xue , L. B. Xiong , H. L. Ma , C. Xue , Y. C. Zhang , J. B. Li , M. T. Chen , Z. S. Li , T. Y. Liu , Z. L. Zhang , P. Dong , Y. H. Li , K. Yao , Z. Q. Hu , X. F. Chen , J. X. Luo , Y. H. Lei , P. Y. Liang , Z. Z. Liu , L. Qi , W. F. Xu , Z. G. Cao , N. H. Chen , X. Li , X. N. Sheng , G. H. Luo , B. K. Shi , et al., Cancer Cell 2025, 43, 970. 40215977 10.1016/j.ccell.2025.03.023

[61] L. Malle , R. S. Patel , M. Martin‐Fernandez , O. J. Stewart , Q. Philippot , S. Buta , A. Richardson , V. Barcessat , J. Taft , P. Bastard , J. Samuels , C. Mircher , A. S. Rebillat , L. Maillebouis , M. Vilaire‐Meunier , K. Tuballes , B. R. Rosenberg , R. Trachtman , J. L. Casanova , L. D. Notarangelo , S. Gnjatic , D. Bush , D. Bogunovic , Nature 2023, 615, 305. 36813963 10.1038/s41586-023-05736-y PMC9945839

