# Supplementary material for: Spatial Transcriptional Dynamics of CD74⁺ B Cells in Tertiary Lymphoid Structures Drive Immune Evolution in Penile Squamous Cell Carcinoma
Source: Adv Sci (Weinh). 2025 Oct 20;12(44):e09742. doi: 10.1002/advs.202509742 (PMC12667480; doi:10.1002/advs.202509742)
Supplement: Supplementary file 1 — Supporting Information [file ADVS-12-e09742-s001.docx]

**Supporting Information for**

Spatial Transcriptional Dynamics of CD74⁺ B Cells in Tertiary Lymphoid Structures Drive Immune Evolution in Penile Squamous Cell Carcinoma

Ting Xue^1, 2 †^, Chuangzhong Deng^1,3, †^, Jingya Liu^4, †^, Ru Yan^5^, Jing Li^6^, Xiheng Hu^7,8,9,10^, Xueying Li^11^, Xiao Xiao^12^, Jietian Jin^12^, Hongzhen Tang^4^, Desi Chen^13^, Zihan Zuo^14^, Yujie Liang^15^, Dongbin Wang^16^, Bonan Chen^17,18,19*^, Hui Han^2,20, *^, Zaishang Li^13,21,22 *^

Correspondence to: bonanchen@cuhk.edu.hk, hanhui@sysucc.org.cn, lizaishang2017@163.com

**This PDF file includes:**

Supplementary Figures. S1 to S9

**
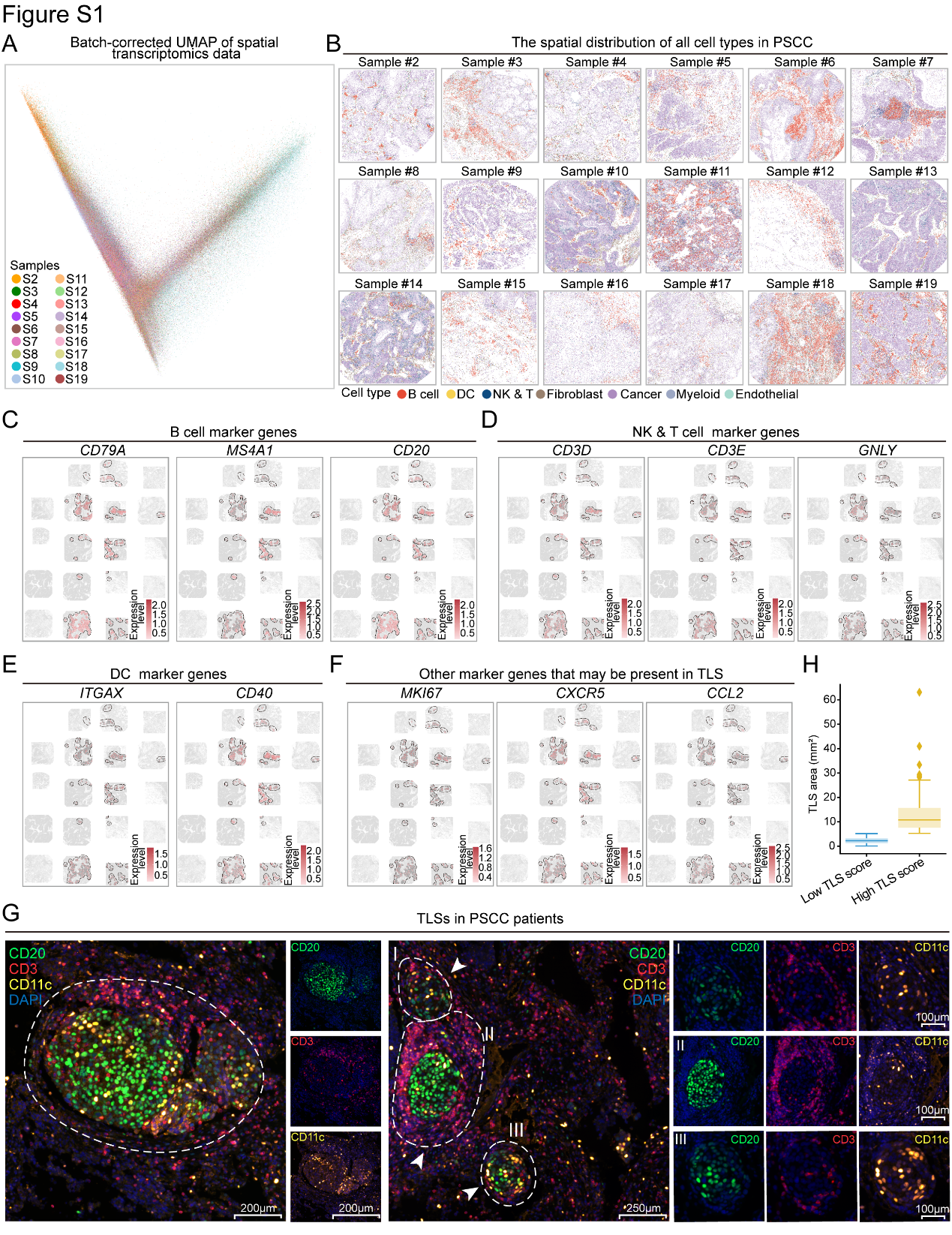
**

**Figure S1.** Localization of TLSs in PSCC patients, related to Figure 1. A) Batch-corrected UMAP of the spatial transcriptomic data. B) In situ distribution of all the cells in the PSCC samples, with different coloured dots representing different cell types. C-E) Expression levels of biomarker genes for B cells (*CD79A*, *MS4A1*, and *CD20*), NK and T cells (*CD3D*, *CD3E*, and *GNLY*), and dendritic cells (*ITGAX* and *CD40*). Redder colours indicate higher gene expression, and dashed circles indicate TLS regions. F) Expression levels of potential TLS biomarkers, including proliferating B cells (*MKI67*), T follicular helper cells (*CXCR5*), and the chemokine *CCL2*. Redder colours indicate higher gene expression, and dashed circles indicate TLS regions. G) Representative mIHC staining of CD20 (green), CD3 (red), and CD11c (yellow) in TLS regions of PSCC tissue sections. The dashed areas highlight dense structures composed of CD20⁺, CD3⁺, and CD11c⁺ cells, which were identified as TLSs. H) Box plots showing the distribution of TLS area (mm²) for patients in the high and low TLS groups. Patients were categorized into these groups based on whether their cumulative TLS area exceeded the median threshold (5.16 mm²) for the entire cohort of 152 patients.


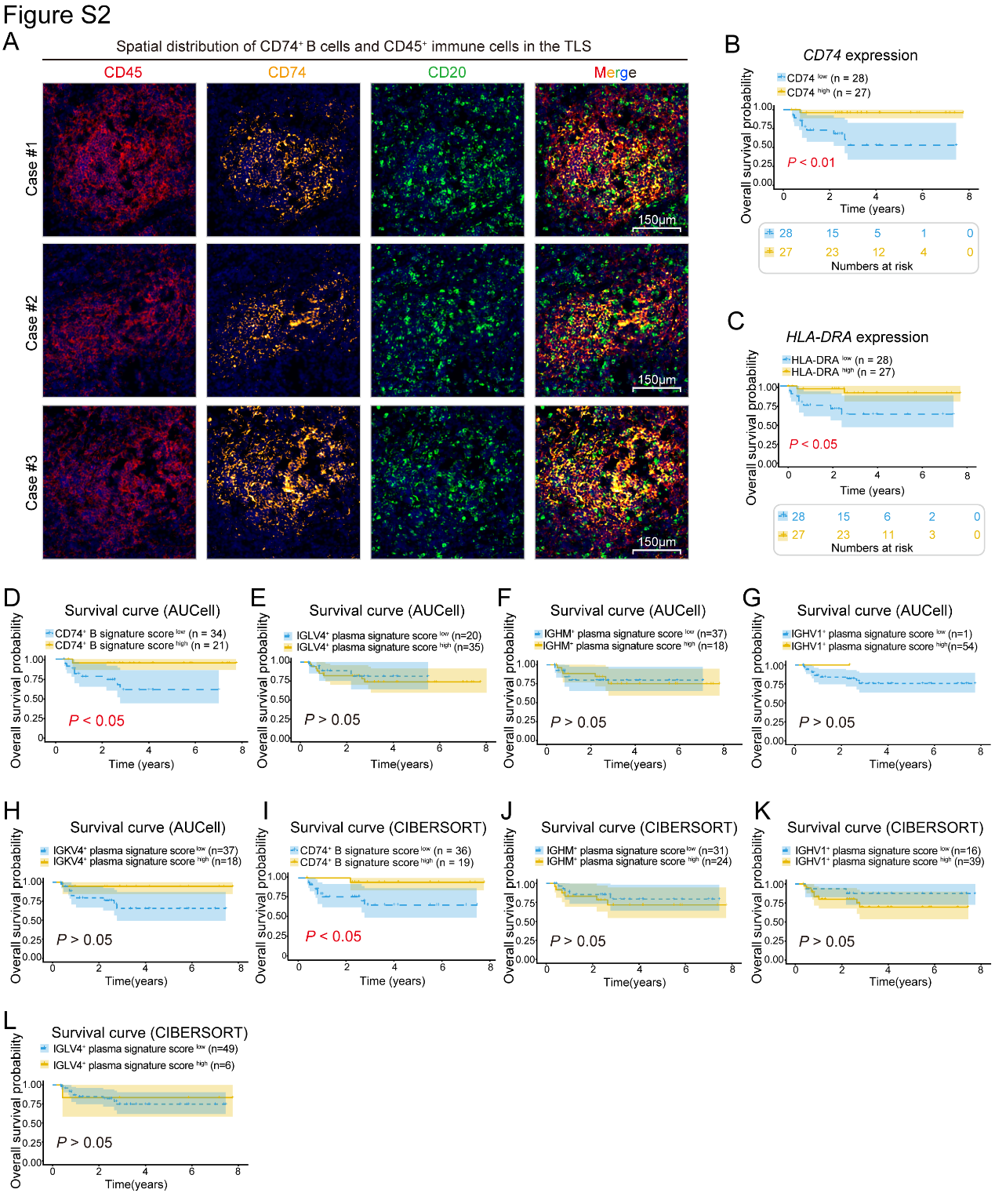


**Figure S****2.** Localization of CD74⁺ B cells and survival analysis of B-cell subtypes in patients with PSCC on the basis of the bulk RNA sequencing data, related to Figure 2. A) Representative mIHC images showing CD45 (red), CD74 (orange), and CD20 (green) in TLSs from three PSCC patient samples (Scale bar = 150 μm). B, C) Survival analysis of PSCC patients stratified by high and low expression levels of CD74 (n = 55, *P* < 0.01) and HLA-DRA (n = 55, *P* < 0.05). D) Survival analysis of CD74⁺ B cells in PSCC patients based on AUCell signature scores (n = 55, *P* < 0.05). E–H) Survival analysis of patients with PSCC on the basis of AUCell signature scores considering IGLV4⁺, IGHM⁺, IGHV1⁺, and IGKV4⁺ plasma cells (n = 55, *P* > 0.05). I) Survival analysis of patients with PSCC on the basis of CIBERSORT signature scores considering CD74⁺ B cells (n = 55, *P* < 0.05). J–L) Survival analysis of patients with PSCC on the basis of CIBERSORT signature scores considering IGHM⁺, IGHV1⁺, and IGLV4⁺ plasma cells (n = 55, *P* > 0.05).


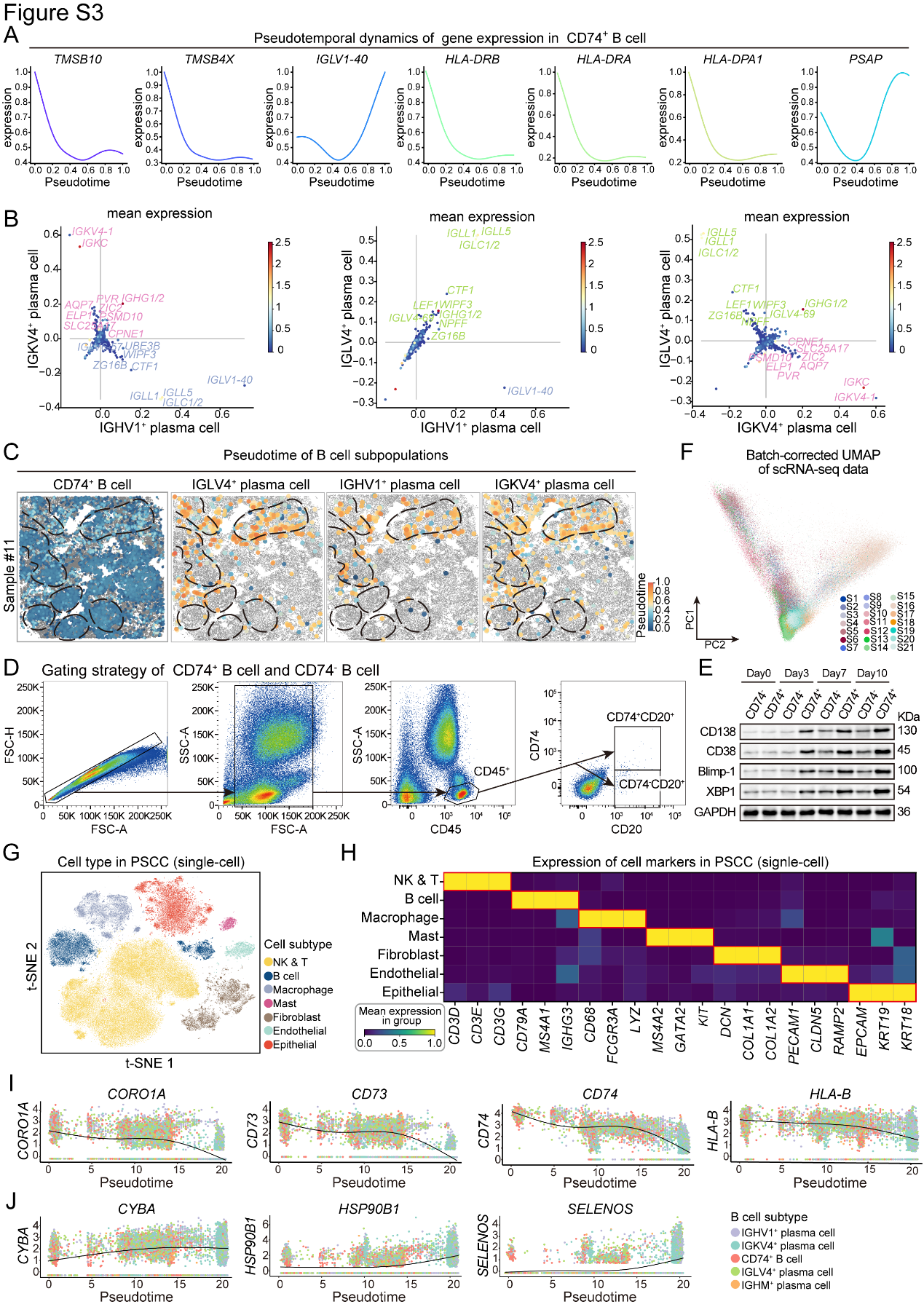


**Figure S3.** Dynamic pseudotime analysis of the CD74⁺ B-cell population, related to Figure 3. A) Dynamic pseudotime analysis of the expression of the *TMSB10, TMSB4X, IGLV1-40, HLA-DRB, HLA-DRA, HLA-DPA1,* and *PSAP* genes in CD74⁺ B cells. B) Critical regulatory genes driving the development of *IGHV1⁺*, *IGKV4⁺*, and *IGLV4⁺* plasma cells. C) Pseudotime developmental evolution of B-cell subtypes across patient samples, with colours transitioning from light blue to orange-red indicating progression from the early to late developmental stages. The dashed circles indicate TLS regions. D) Gating strategy for CD74⁻ B cells and CD74⁺ B cells sorted from the tissues of six PSCC patients. E) Representative images of protein expression levels for CD138, CD38, Blimp-1, and XBP1 in CD74⁻ B cells and CD74⁺ B cells on Day 0, 3, 7, and 10 of culture. F) Batch-corrected UMAP plot of the scRNA-seq data. G) t-SNE plot of cell types in PSCC tissue, with different coloured dots representing different cell types. H) Expression levels of cell type-specific biomarkers, including *CD3D*, *CD3E*, and *CD3G* for NK and T cells; *CD79A*, *MS4A1*, and *IGHG3* for B cells; *CD68*, *FCGR3A*, and *LYZ* for macrophages; *MS4A2*, *GATA2*, and *KIT* for mast cells; *DCN*, *COL1A1*, and *COL1A2* for fibroblasts; *PECAM1*, *CLDN5*, and *RAMP2* for endothelial cells; and *EPCAM*, *KRT19*, and *KRT18* for epithelial cells. Yellower colours indicate higher mean gene expression levels. I) Pseudotime expression trends in the expression of immune-regulatory genes (*CORO1A* and *CD73*) and antigen presentation-related genes (*CD74* and *HLA-B*) in B-cell subpopulations. J) Pseudotime expression trends in the expression of apoptosis- and stress response-related genes (*CYBA*, *HSP90B1*, and *SELENOS*) in B-cell subpopulations. The data in D-H were derived from the scRNA-seq data of PSCC patients.


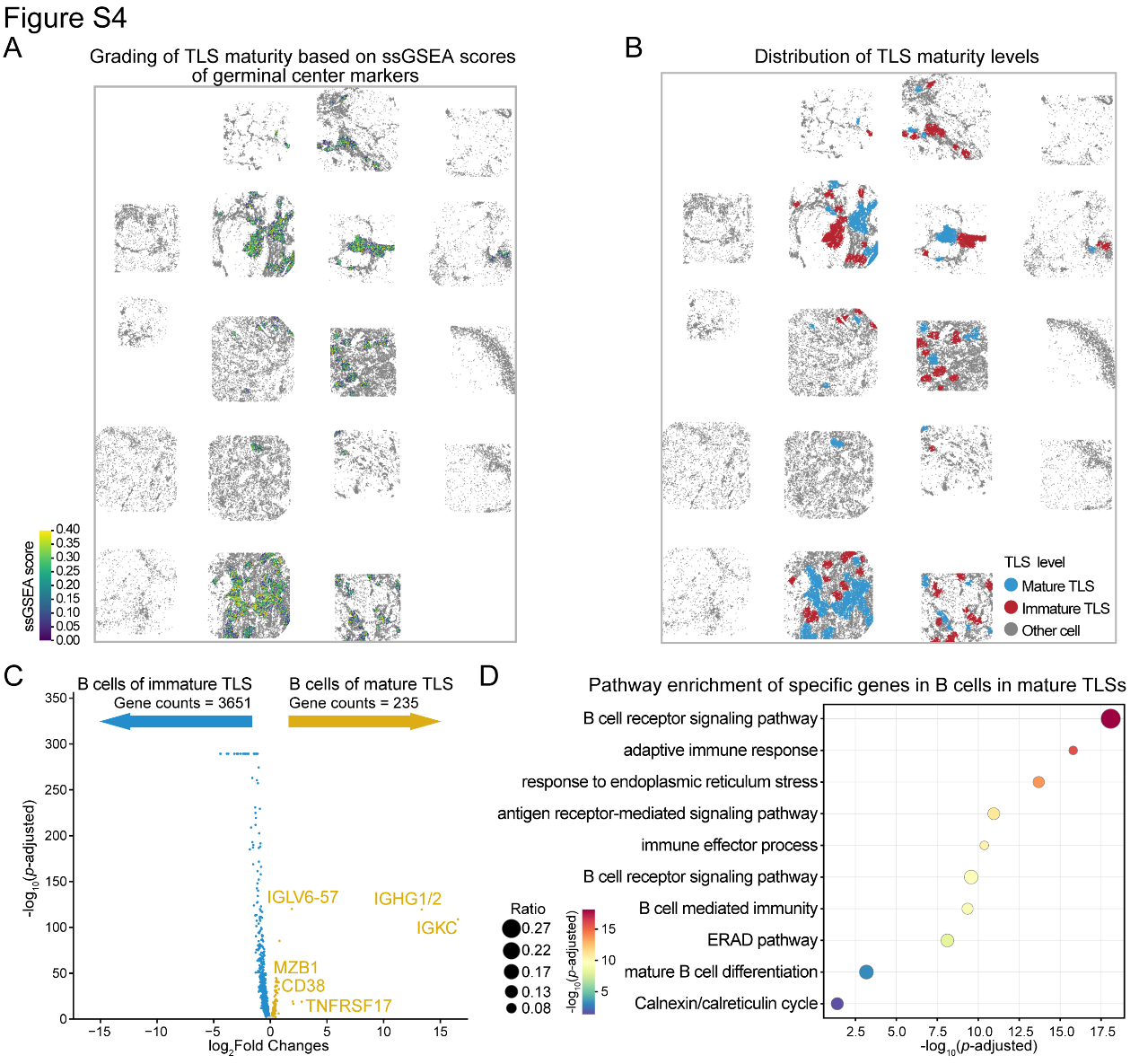


**Figure S4.** Functional plasma cell differentiation and a stronger antigen-driven immune response are present in mature TLSs, related to Figure 4. A) Based on ssGSEA scores of a series of germinal center markers (*BCL6*, *AICDA*, *CD38*, *ICOS*, *CXCR5*, *CXCL13*), TLS maturity was scored, with colors ranging from green to yellow indicating higher TLS scores. B) TLS with scores above the cohort median were classified as mature, while those below the median were classified as immature. Blue represents mature TLSs, red represents immature TLSs, and grey represents other cells. C) Volcano plot of differential genes in B cells from mature versus immature TLSs. D) Pathway enrichment bubble plot of specifically upregulated genes in B cells from mature TLSs compared to those from immature TLSs, where the size of the points indicates the gene ratio, and the redder the colour, the higher the significance of the pathway.

**
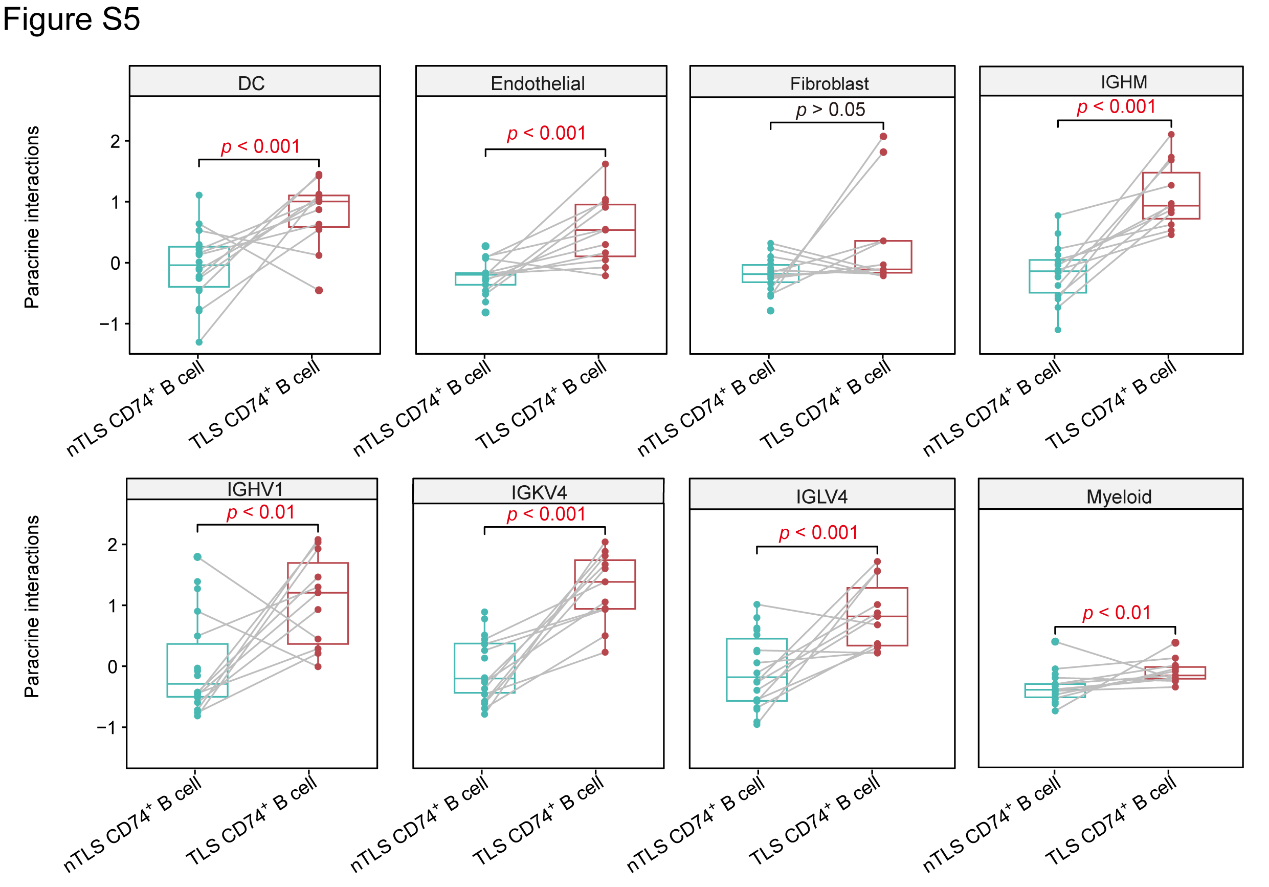
Figure S5.** Paracrine interactions of various cell types with CD74⁺ B cells in TLSs and nTLSs, related to Figure 5. The boxplots display the paracrine interaction scores between CD74⁺ B cells and different cell types in TLSs and nTLSs, with *P* < 0.01 and *P* < 0.001 indicating significant differences and *P* > 0.05 indicating no statistically significant difference. Two-sided Welch t-test (unpaired), n =18.


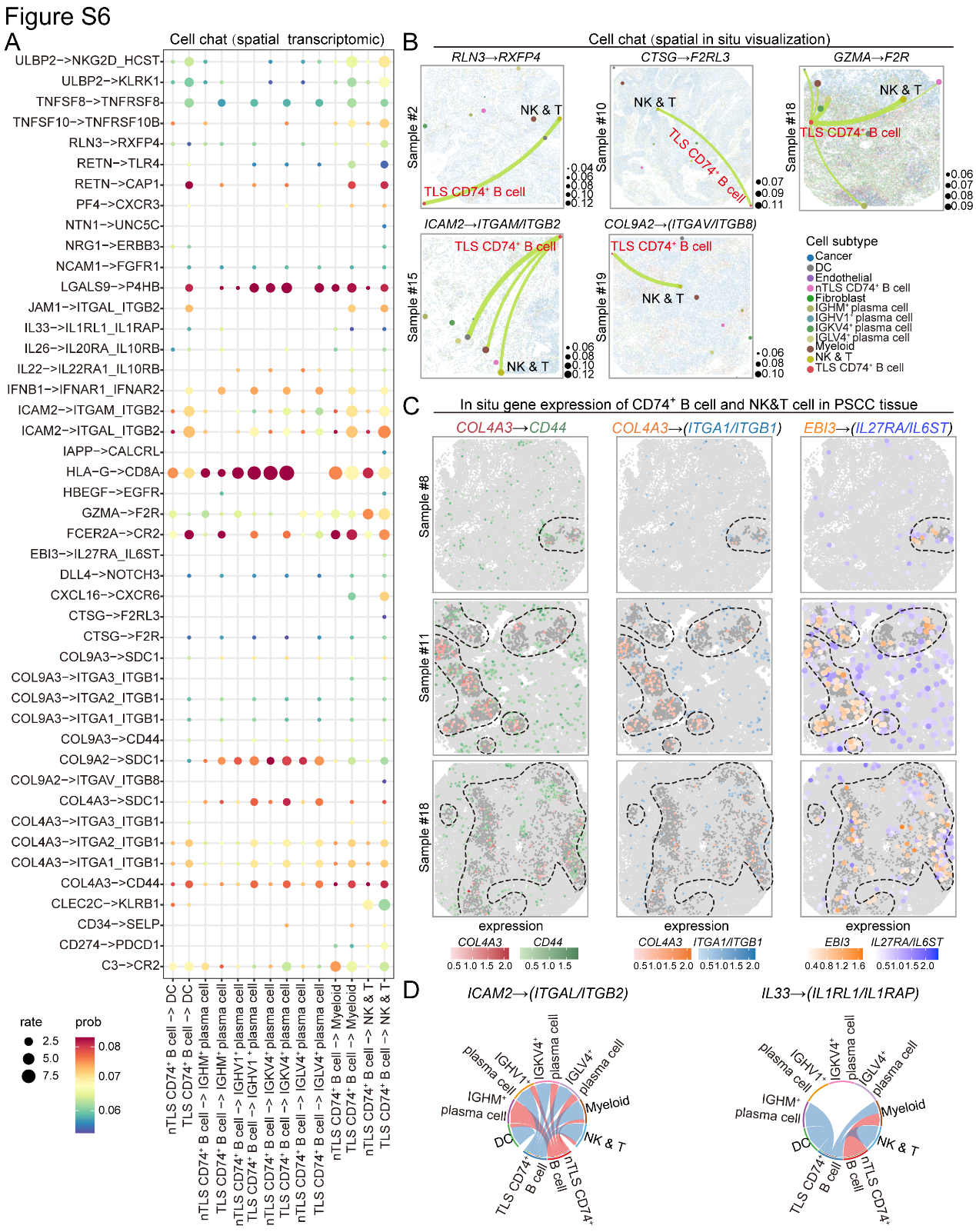


**Figure S6.** Analysis of CD74⁺ B cell communication in TLSs and nTLSs and an in situ visualization of ligand‒receptor pairs, related to Figure 5. A) Dot plot showing ligand‒receptor interactions between CD74⁺ B cells in TLSs or nTLSs and other cells. The dot size represents the communication ratio, while increasing redness indicates a greater likelihood of interaction. B) Spatial in situ communication plots depicting ligand‒receptor interactions between CD74⁺ B cells in TLSs and NK and T cells across PSCC samples. The dot size indicates the proportion of cells relative to the total population, and the line thickness reflects the interaction strength. C) Coexpression of key ligand‒receptor pairs between CD74*⁺* B cells and NK and T cells in PSCC tissue. *COL4A3* and *EBI3* are expressed by CD74*⁺* B cells, whereas *CD44*, *ITGA1/ITGB1*, and *IL27RA/IL6ST* are expressed by NK and T cells. The dashed areas indicate TLSs, and darker colours represent higher expression levels. D) Circular diagrams showing ligand‒receptor pair communication between cells.


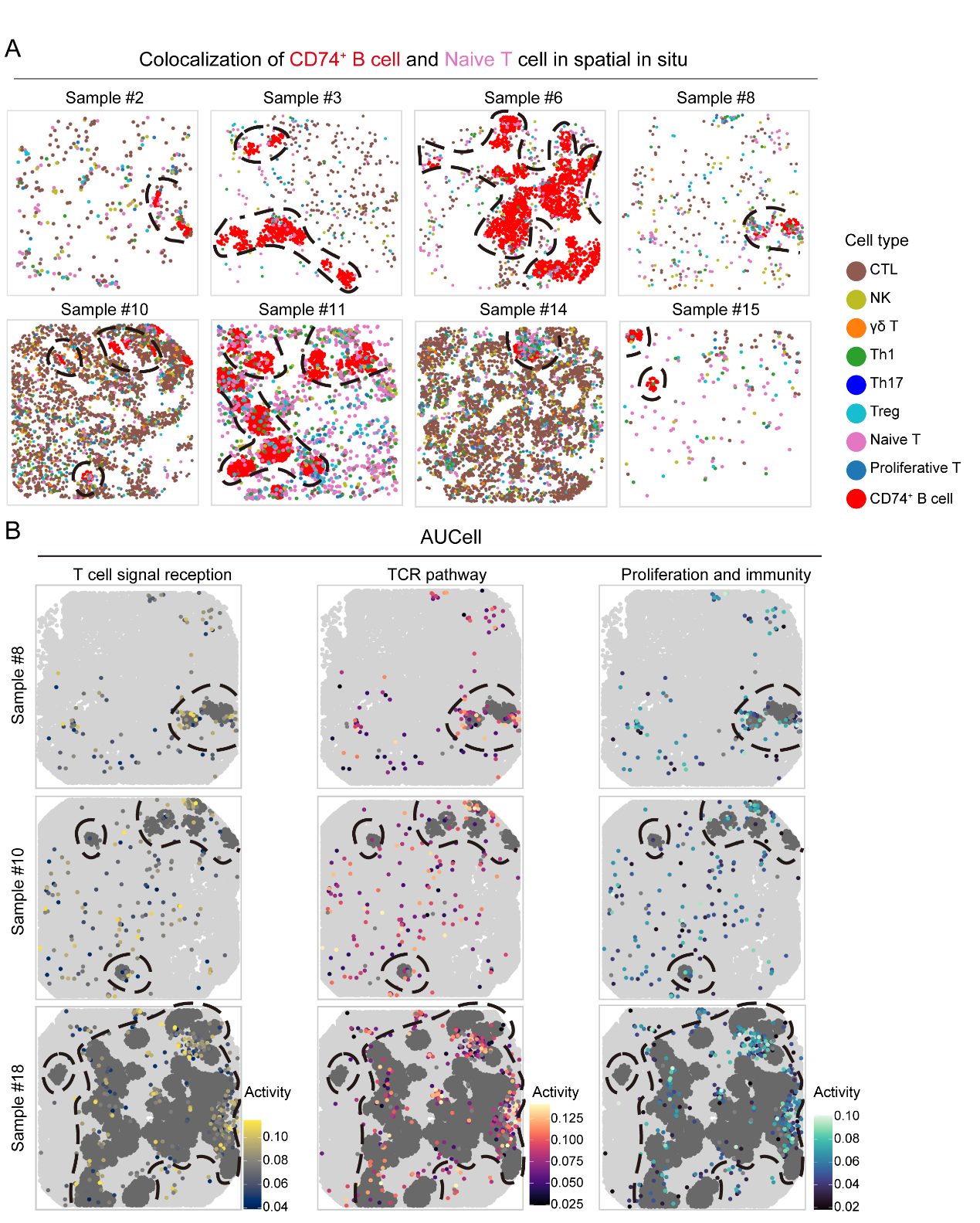


**Figure S7.** Spatial distribution and AUCell analysis of naive T-cell activity in PSCC tissues, related to Figure 6. A) Spatial distribution of various cell types in PSCC tissues, with dashed boxes highlighting the TLS regions. B) Representative AUCell analysis of naive T-cell pathway activity based on MSigDB gene sets. Lighter colours indicate higher activity, and grey areas represent TLS regions.


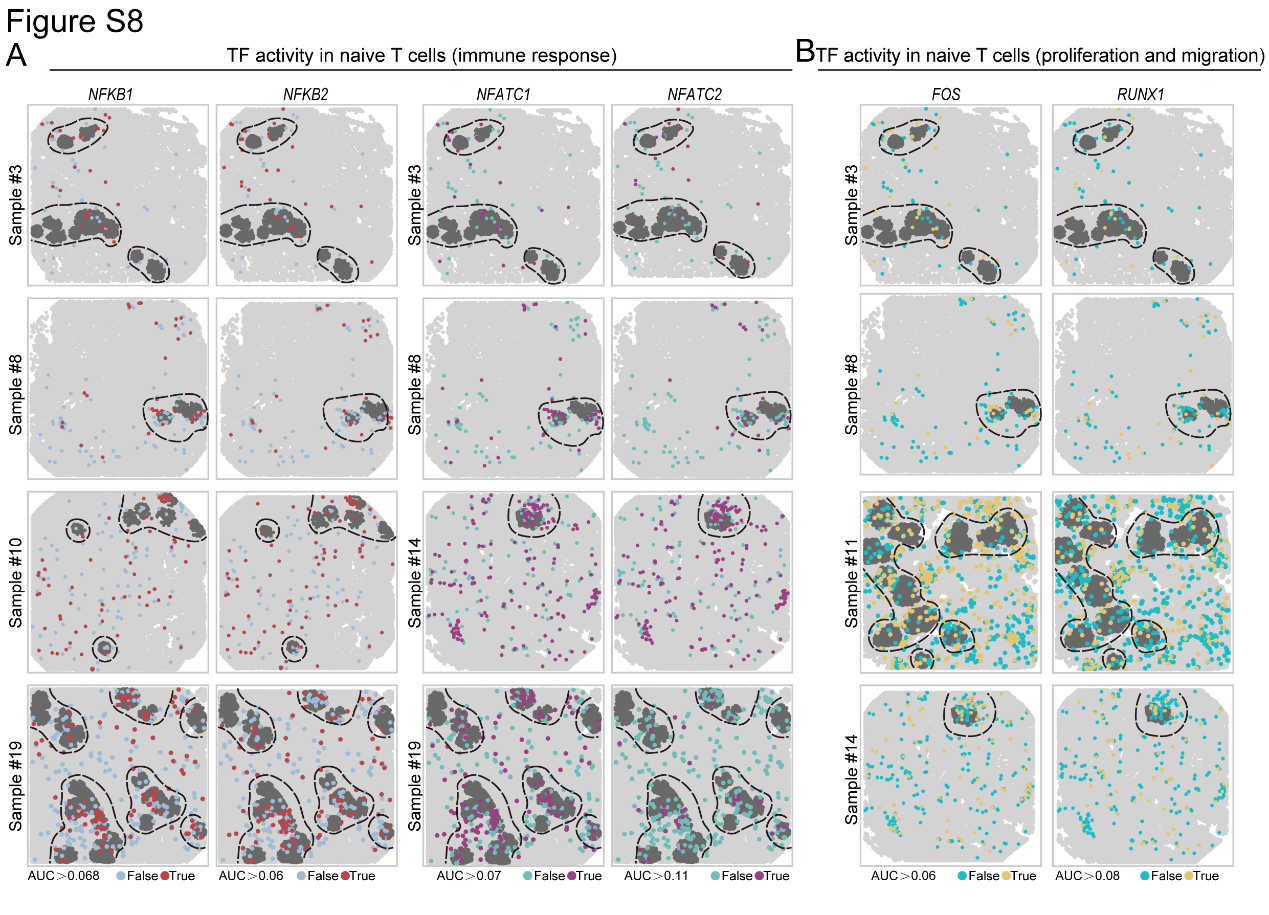


**Figure S8.** Transcription factor activity in naive T cells within the TLSs of PSCC tissues, related to Figure 7. A, B) In situ expression of transcription factors related to immunity, proliferation, and migration in naive T cells. "True" indicates transcription factor activation, whereas "false" indicates inactivity. The dashed boxes mark the TLS regions.

**
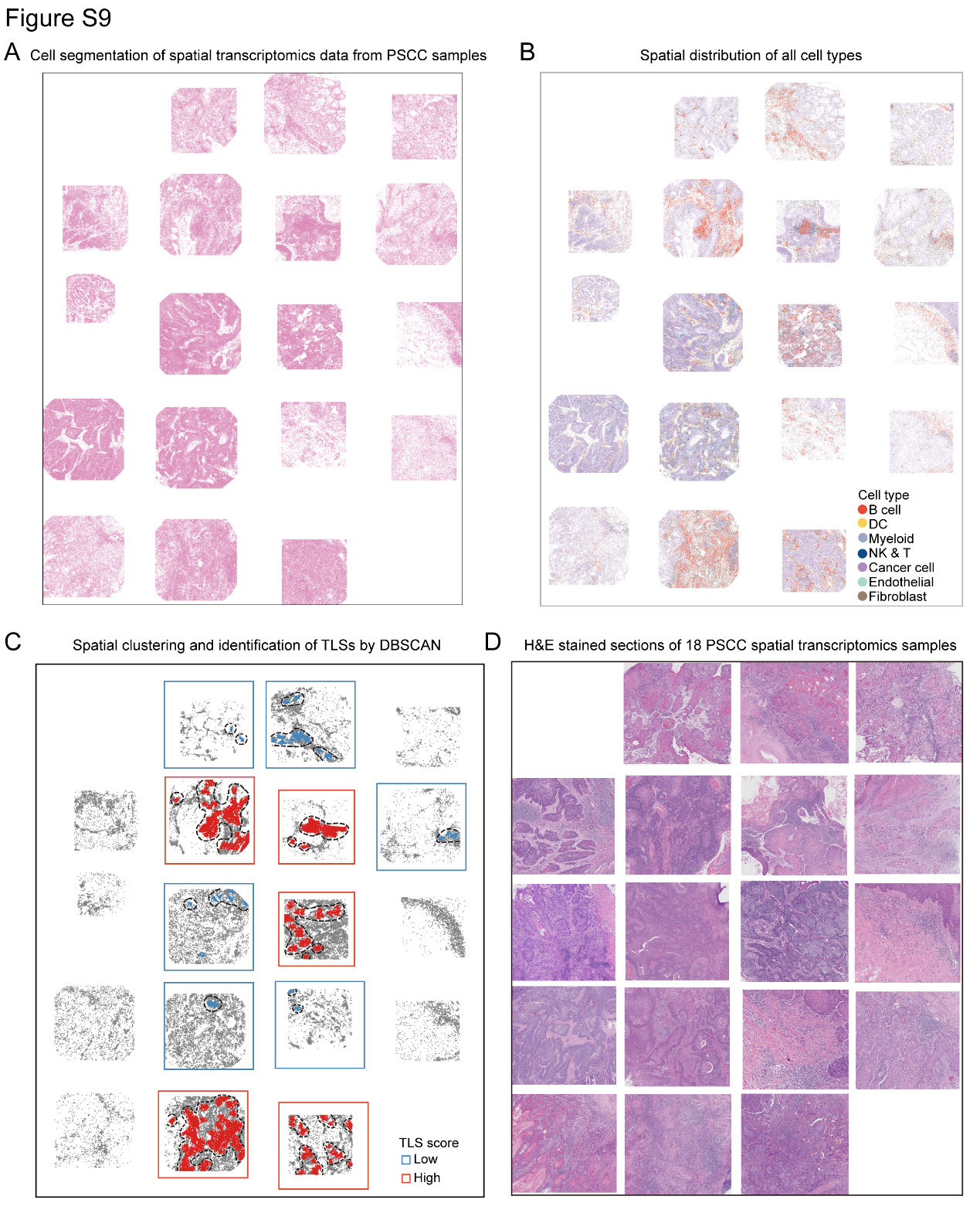
**

**Figure S9.** Definition of TLS structure, related to the method 'Identification of the TLS region'. A) Cell segmentation images of 18 spatial transcriptomics samples. B) In situ distribution of cell types. C) TLS-like regions defined using the DBSCAN algorithm based on the aggregation of B cells, NK cells, T cells, and DCs, with blue boxes indicating low-scoring TLS regions and red boxes indicating high-scoring TLS regions. D) H&E staining schematic of spatial transcriptomics samples.
